# RPLP2 Mediates the Beneficial Effects of Exercise on Stress Resistance Through Muscle–Brain Communication

**DOI:** 10.1002/advs.76479

**Published:** 2026-07-09

**Authors:** Peiyu Luo, Wei Wu, Huan Peng, Dan He, Yuxi Guo, Boyue Zhao, Xiaodan Wang, Li Ma, Yuhang Qin, Yifang Zhai, Lixia Zhuo, Ying Zhang, Yijie Guo, Linlin Jing, Fangyao Chen, Erfei Zhang, Wei Wang, Xiancang Ma, Yan Li

**Affiliations:** ^1^ Department of Psychiatry The First Affiliated Hospital of Xi'an Jiaotong University Xi'an Shaanxi China; ^2^ Department of Anesthesiology The First Affiliated Hospital of Xi'an Jiaotong University Xi'an Shaanxi China; ^3^ Center For Brain Science The First Affiliated Hospital of Xi'an Jiaotong University Xi'an Shaanxi China; ^4^ School of Public Health Xi'an Jiaotong University Health Science Center Xi'an Shaanxi China; ^5^ Department of Anesthesiology The Affiliated Hospital of Yan'an University Yan'an Shaanxi China; ^6^ Shaanxi Belt and Road Joint Laboratory of Precision Medicine in Psychiatry The First Affiliated Hospital of Xi'an Jiaotong University Xi'an Shaanxi China; ^7^ Shaanxi Provincial Key Laboratory of Biological Psychiatry The First Affiliated Hospital of Xi'an Jiaotong University Xi'an China

**Keywords:** anxiety disorders, exercise, muscle–brain communication, neurogenesis, ribosome assembly, RPLP2, stress resistance

## Abstract

Anxiety disorders pose a considerable burden on public health. While exercise has been increasingly established as a viable strategy for preventing and alleviating anxiety symptoms, the underlying molecular mechanisms of this action remain elusive. We previously conducted an 8‐week randomized controlled trial which suggested that exercise not only significantly reduced anxiety levels but also triggered the release of acidic ribosomal protein P2 (RPLP2) from peripheral tissues into the bloodstream. These elevated circulating RPLP2 levels showed an inverse association with anxiety severity in patients, suggesting a potential therapeutic role for the protein. Further experiments in mice confirmed that skeletal muscle–derived RPLP2 exerts its anxiolytic effects by facilitating hippocampal neurogenesis. Mechanistically, RPLP2 facilitates ribosomal localization and increases the efficiency of ribosomal subunit assembly, thereby increasing local protein synthesis and supporting the development and maturation of newly generated neurons. The successful maturation of these neurons, in turn, promotes morphological and functional synaptic plasticity, which is crucial for the integration and functional maturation of newborn neurons into hippocampal circuits. Collectively, our findings reveal a previously unknown muscle–brain axis mediated by RPLP2, offering mechanistic evidence for exercise‐induced stress resistance.

## Graphical abstract

A novel exercise‐inducible myokine acidic ribosomal protein P2 (RPLP2), initially identified from human trials, is presented here, whose circulating levels negatively correlate with clinical anxiety severity. Muscle‐derived RPLP2 enhances hippocampal ribosomal assembly and adult neurogenesis to rescue stress‐induced anxiety deficits. These findings establish a previously unrecognized peripheral muscle–brain regulatory axis and identify RPLP2 as a promising therapeutic target for anxiety disorders.

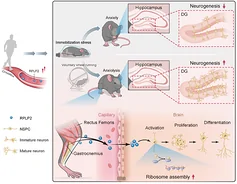

## 1 Introduction

Anxiety disorders represent a major category of psychiatric illness with high global prevalence [1] and, in addition to significantly impacting physical health, generate substantial social and economic costs [2]. Current treatments are based primarily on psychiatric medications, especially selective serotonin reuptake inhibitors (SSRIs), which act by modulating serotonin reuptake [3, 4]. However, a substantial percentage of patients, ranging from 20% to 30%, does not respond to these treatments [5], and many experience severe adverse effects such as jitteriness, nausea, fatigue, and headache [6, 7]. These adverse events highlight the urgent need to better clarify the biological mechanisms underlying anxiety disorders and to explore innovative treatment strategies.

Physical exercise is increasingly recognized as a safe and effective nonpharmacological approach for anxiety symptoms [8, 9]. In addition to its anxiety‐reducing benefits, exercise offers a wide array of advantages for brain function, including greater cognitive performance [10, 11] and improved memory [12, 13]. Furthermore, exercise exerts protective effects on peripheral organs, including the heart [14], liver [15], skeletal muscles [16], and adipose tissue [17]. These systemic benefits indicate that exercise‐associated anxiolysis may be mediated by complex crosstalk between peripheral tissues and the central nervous system. Notably, skeletal muscle, traditionally regarded as a structural and metabolic organ, is now recognized as an endocrine organ that releases signaling molecules called myokines during exercise [18, 19]. Despite the increasing recognition of myokines as crucial mediators of exercise‐induced benefits, the contribution of muscle tissues in alleviating anxiety through exercise remains insufficiently understood.

Ribosomal proteins are vital components of the translational machinery and play essential roles in ribosome assembly and function [20, 21, 22]. Ribosomes are fundamental for protein synthesis, and their activity is tightly regulated by the dynamic demands of cellular processes [23]. Although ribosomal proteins are traditionally considered intracellular components, multiple studies have revealed that a subset of them can be secreted and may function in the extracellular environment [24, 25, 26]. These observations raise the possibility that specific ribosomal‐selected ribosomal proteins are involved in interorgan communication under physiological conditions such as those induced by exercise. The acidic ribosomal P protein family is essential for ribosome assembly and efficient protein synthesis [20, 21, 22].

In this study, we discovered that acidic ribosomal protein P2 (RPLP2) contributes to the morphological and functional maturation of newborn hippocampal neurons by increasing the efficiency of ribosomal subunit assembly. Specifically, muscle‐derived RPLP2 contributes to the anxiolytic actions of exercise through a muscle‐to‐brain pathway. The results of this study reveal a previously unrecognized mechanism underlying exercise‐induced stress resistance and highlight RPLP2 as a potential therapeutic candidate for treating anxiety disorders.

## 2 Results

### 2.1 Exercise Relieves Anxiety and Increases Circulating RPLP2 Levels

During the COVID‐19 pandemic in 2021, we conducted a randomized controlled trial (RCT) (Figure S1), and the results showed no significant differences in sex, education background, age, weight, height, body mass index (BMI), total weekly physical activity, or sitting time at baseline between the exercise and control groups (Table S1). We also found that moderate aerobic exercise significantly alleviated anxiety symptoms in a group of participants, particularly at the end of the 8‐week intervention (Table S2). These behavioral improvements prompted us to further explore the circulating molecular changes that may underlie these anxiolytic effects of exercise.

To investigate exercise‐induced alterations in serum proteins and identify candidate targets that may be associated with exercise‐related alleviations of anxiety symptoms, we performed comprehensive proteomic profiling of serum samples from runners and nonrunners (who served as the control group) after an 8‐week intervention, followed by correlation analyses and machine learning–based prioritization to identify candidate proteins (Figure 1A). A total of 67 proteins were significantly differentially expressed between the exercise and control groups, including 30 upregulated and 37 downregulated proteins (Figure 1B and Figure S2A). We next conducted correlation analyses between the abundance of the 67 differentially expressed proteins (DEPs) and the Zurich Self‐Rating Anxiety Scale (SAS) [27] scores of the participants. The results revealed that the abundance of five DEPs—including hypoxia‐upregulated protein 1 (HYOU1), 60S RPLP2, protease serine 2 preproprotein (PRSS2), zymogen granule membrane protein (ZG16), and poly(A) polymerase alpha (PAPOLA)—was negatively correlated with the SAS score (Figure S2B–F), whereas the abundance of four DEPs—including filaggrin (FLG), ribosomal protein L15 (RPL15), chordin‐like protein 2 (CHRDL2), and keratin 4 (KRT4)—was positively correlated with the SAS score (Figure S2G–J). In addition, leveraging a proteomic dataset, we developed a machine learning model to identify exercise‐related protein markers. Through an incremental feature selection (IFS) strategy combined with an ensemble voting classifier that integrates three complementary base models—logistic regression (LR), support vector machine (SVM), and random forest (RF)—we identified an optimal feature subset (Figure S2K–M). The model achieved an area under the curve (AUC) of 0.833 in predicting exercise status in the independent test cohort (Figure 1C). Critically, among all the candidates, RPLP2 was retained in the optimal subset and was classified as a DEP, and its level was inversely correlated with the SAS score (Figure 1D). On its own, the RPLP2 level achieved an AUC of 0.921 in the independent test cohort (Figure 1E).

**FIGURE 1:**
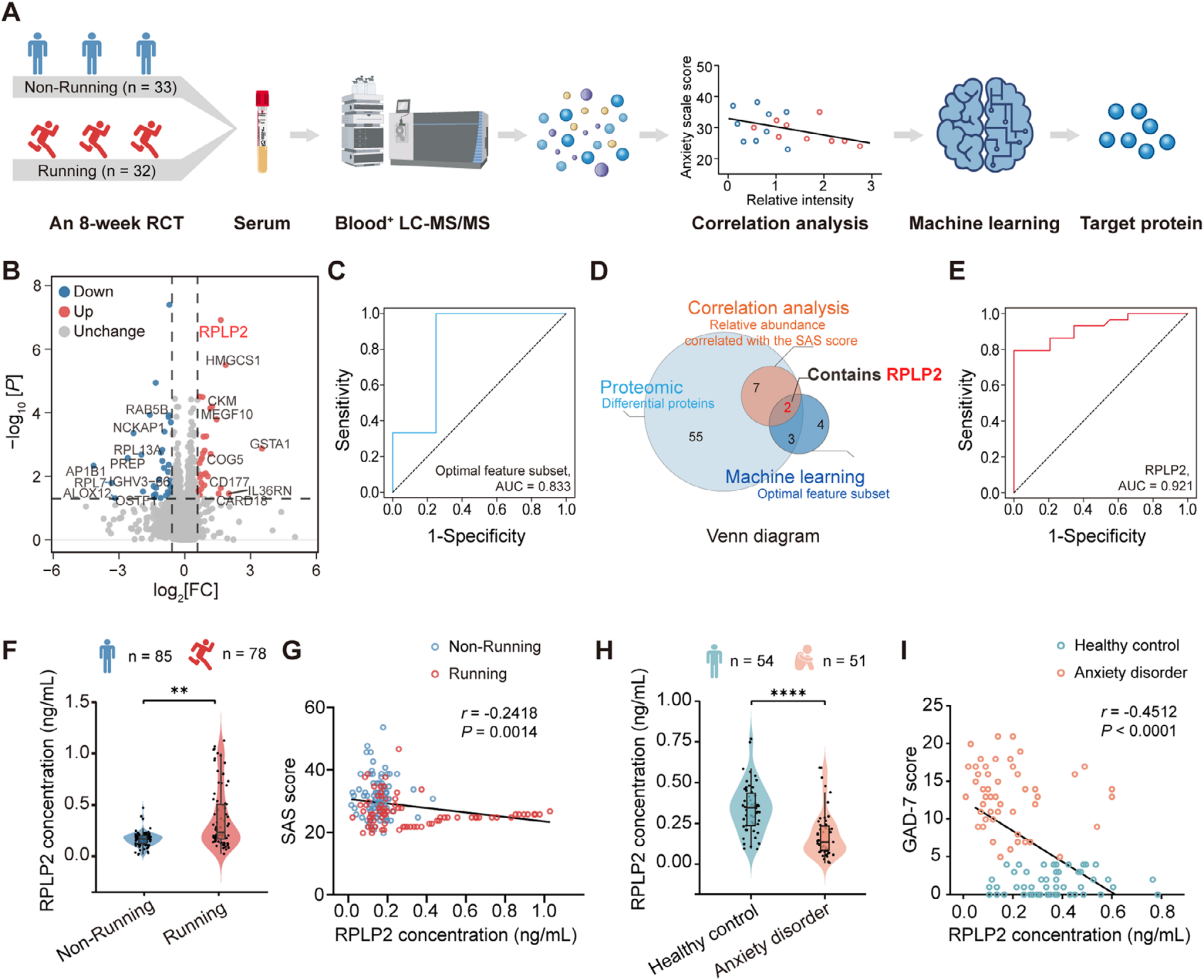
The exercise‐induced serum RPLP2 level is inversely correlated with anxiety severity. (A) Workflow for processing the serum proteomic data, including participant intervention, LC‒MS/MS analysis, machine learning analysis, and correlation analysis. (B) Volcano map of the DEPs. The cutoff for the fold change (FC) was set to 1.5, and the *p*‐value was set to 0.05. The 20 proteins with the lowest *p*‐values are annotated. (C) Receiver operating characteristic (ROC) curve and area under the curve (AUC) for the protein‐based algorithm using the optimal feature subset in distinguishing runners from nonrunners in the independent test cohort. (D) Venn diagram of screening results from proteomics, correlation analyses, and machine learning analyses. (E) ROC curve and AUC of RPLP2 are independently distinguishing runners from nonrunners in the independent test cohort. (F) Serum levels of RPLP2 in runners and nonrunners after an 8‐week exercise intervention (*n* = 85 individuals in the nonrunning group; *n* = 78 individuals in the running group). (G) The serum RPLP2 level is negatively associated with the severity of anxiety, as measured by the SAS, *r* = −0.2418, *p* = 0.0014 (*n* = 85 individuals in the nonrunning group and *n* = 78 individuals in the running group). (H) Serum levels of RPLP2 in patients with anxiety disorders and healthy controls (*n* = 54 healthy controls; *n* = 51 patients with anxiety). (I) The serum RPLP2 level is negatively associated with the severity of anxiety, as measured by the GAD‐7 scale (*r* = −0.4512; *p* < 0.0001; *n* = 54 healthy controls; and *n* = 51 patients with anxiety). DEPs: differentially expressed proteins; SAS: Zurich Self‐Rating Anxiety Scale; GAD‐7: generalized anxiety disorder‐7 scale. Statistical analyses, including two‐tailed unpaired *t*‐tests with Welch's correction and Pearson correlation analysis, were performed with GraphPad Prism 9. **p < 0.01 and ****p < 0.0001.

RPLP2, an acidic P‐protein family member located in the eukaryotic 60S ribosomal subunit, forms a P‑protein stalk with RPLP0 and RPLP1, and is involved in mediating translation. The protein contains 115 amino acids and has an approximate molecular mass of 15 kDa [22]. Given these characteristics, we hypothesized that RPLP2 can serve as a biomarker for exercise intervention and is involved in the mechanisms through which exercise ameliorates anxiety symptoms. To validate this hypothesis, we performed two population‐based enzyme‐linked immunosorbent assays (ELISAs). First, we assessed serum samples obtained from participants in a previous RCT who had not undergone prior proteomics evaluations. Following the running intervention, we observed that circulating RPLP2 levels were significantly higher in the running group than in the nonrunning group (Figure 1F). Moreover, the 8‐week change in the circulating RPLP2 level was significantly greater in the running group than in the nonrunning group (Figure S2N). After the intervention, the circulating RPLP2 concentration was significantly and negatively correlated with the SAS score at the individual participant level (Figure 1G). Second, we analyzed blood samples from a cohort of patients with anxiety; the baseline demographic and clinical characteristics of this cohort (including sex, age, weight, BMI, body fat percentage, Patient Health Questionnaire‐9 (PHQ‐9) score, and Generalized Anxiety Disorder‐7 (GAD‐7) score), together with the corresponding measures for the control group, are summarized in Table S3. Our findings revealed a reduction in circulating RPLP2 levels among these patients compared with healthy individuals (Figure 1H). Notably, the circulating RPLP2 concentration was inversely correlated with the score on the GAD‐7 scale at the individual level in this second cohort (Figure 1I). Collectively, these data suggest a potential role for RPLP2 in the exercise‐mediated alleviation of anxiety symptoms.

### 2.2 Exercise Increases Circulating RPLP2 Levels and Alleviates Impaired Neurogenesis in Stressed Mice

To investigate whether RPLP2 contributes to the anxiety‐alleviating effects of exercise, we first evaluated its circulating levels in exercising mice. Specifically, the mice were subjected to running on a treadmill at a speed of 10 m min^−1^ for 15, 30, 60, 90, or 120 min, after which their circulating RPLP2 levels were measured by ELISA. A consistent increase in circulating RPLP2 levels was observed with increasing durations of treadmill exercise, with a marked increase evident after 60 min (Figure S3A). As a forced paradigm, treadmill running is accompanied by a potential stress response; thus, we introduced a voluntary wheel‐running model to evaluate the effects of exercise without such stress interference. The mice in the exercise groups had continuous access to a running wheel for 28 days, whereas those in the chronic immobilization stress (CIS) groups were restrained for 2 h daily from day 18 to day 28 (Figure 2A). Analysis of the distance run on the wheel revealed that immobilization stress did not influence voluntary running behaviors (Figure S3B). Assessments of the body weights measured during the experiment revealed that those of the mice that were subjected to CIS and did not participate in the exercise program decreased. This observation aligns with earlier studies indicating that anxiety is associated with weight reduction [28]. Notably, exercise prevented this weight loss in the mice that were exposed to CIS (Figure S3C).

**FIGURE 2:**
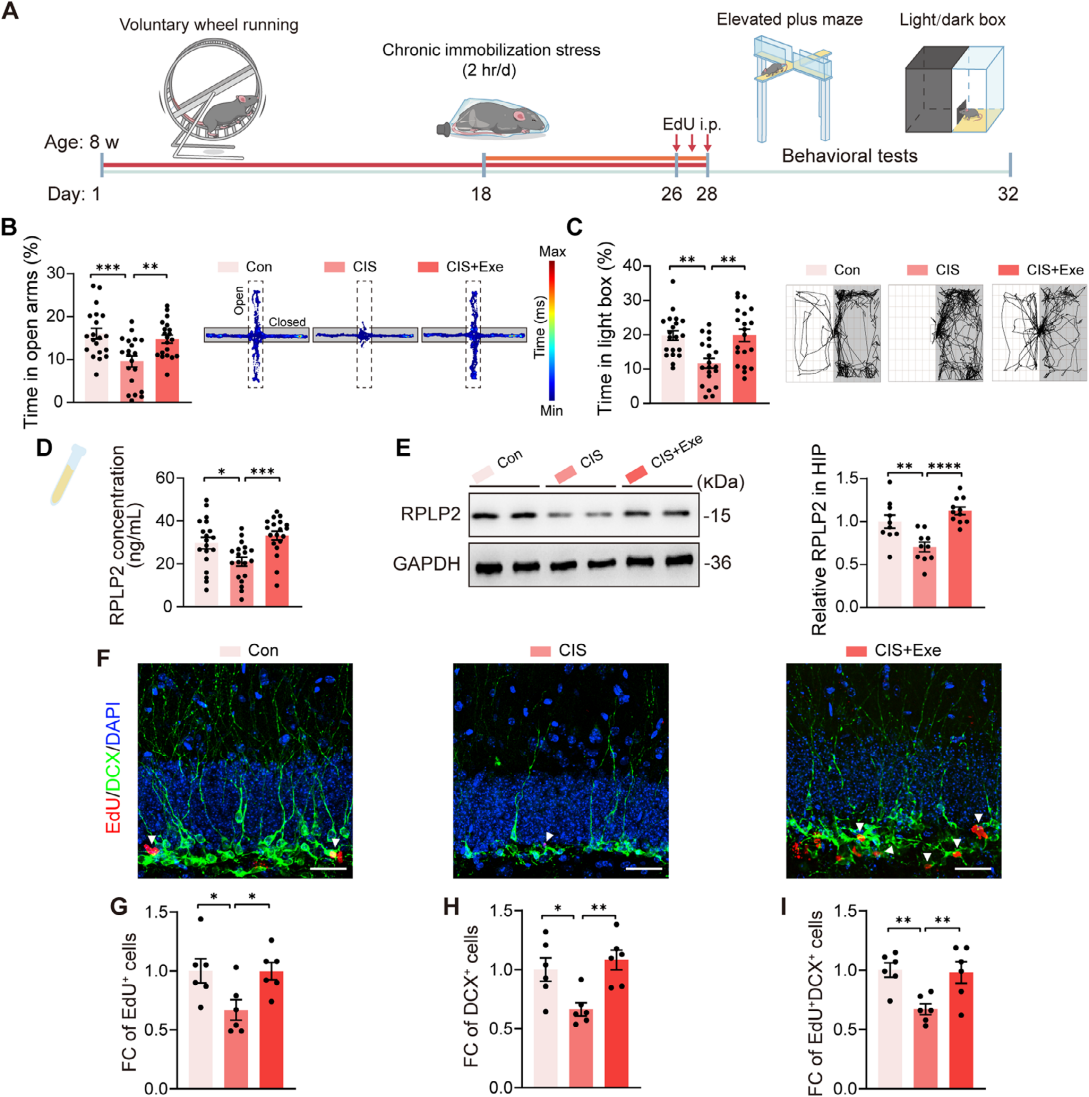
Exercise relieves anxiety, increases RPLP2 levels, and facilitates neurogenesis in mice exposed to CIS. (A) Overview of the study design, including the establishment of the voluntary running wheel exercise model and the CIS‐induced anxiety model as well as several behavioral testing procedures. (B) EPM test results, including the proportion of time spent in the open arms of the maze and representative heatmaps of mouse movement trajectories (*n* = 20 mice per group). (C) Light/dark box test results, including the proportion of time spent in the light box and representative mouse movement traces (*n* = 20 mice per group). (D) Circulating RPLP2 protein levels as quantified with ELISA (*n* = 19 mice per group). (E) Representative immunoblotting bands of RPLP2 from hippocampal tissue and relative protein levels (*n* = 10‒11 mice per group). (F) Representative immunofluorescence images of hippocampal slices stained for EdU and DCX. The white arrows indicate EdU/DCX double‐positive cells. Scale bar = 25 µm. (G–I) Fold change (FC) of (G) EdU+, (H) DCX+, and (I) EdU/DCX double‐positive cells (*n* = 6 mice per group). Con: control, CIS: chronic immobilization stress, Exe: exercise. Statistical analyses, including one‐way analysis of variance (ANOVA) with Tukey's posthoc test and Kruskal–Wallis test with Dunn's posthoc test, were performed using GraphPad Prism 9. All values are presented as the mean ± standard error of the mean (SEM); *p < 0.05, **p < 0.01, ***p < 0.001, and ****p < 0.0001.

Next, anxiety‐related behaviors were evaluated using two established paradigms: the elevated plus‐maze (EPM) test and the light/dark box test. First, no significant differences were observed in the total distance traveled in the EPM among the three groups of mice (Figure S3D). This finding indicates that neither restraint nor exercise intervention affected locomotor activity in the mice. Second, we observed that mice exposed to CIS exhibited marked anxiety‐like behaviors, as evidenced by relatively little time spent in the open arms of the EPM (Figure 2B) and in the illuminated chamber of the light/dark box (Figure 2C). Notably, exercise mitigated these anxiety‐like behaviors in the CIS‐exposed mice (Figure 2B,C). We further examined whether exercise modulates circulating RPLP2 protein levels in mice. ELISA revealed a decrease in circulating RPLP2 levels in CIS mice, whereas the exercise intervention mitigated this reduction (Figure 2D). To further validate the increase in circulating RPLP2 levels observed in exercising mice, we performed serum immunoblotting analysis. Consistent with the ELISA results, the circulating RPLP2 protein levels were greater in serum samples from exercising mice than in those from sedentary controls (Figure S3E). This pattern aligns with our findings in humans, suggesting a conserved mechanism by which exercise alleviates anxiety through the modulation of circulating RPLP2 levels.

Exercise promotes hippocampal neurogenesis [29], a process that is intricately associated with the regulation of anxiety [30, 31, 32]. Ribosomal proteins, including RPLP2, are crucial for supporting protein synthesis during neurogenesis, which in turn is essential for cell proliferation [33]. Therefore, we hypothesized that RPLP2 plays a role in exercise‐induced neurogenesis. To test this hypothesis, we analyzed hippocampal RPLP2 expression and found that exercise increased hippocampal RPLP2 levels in mice exposed to CIS (Figure 2E). We subsequently labeled proliferating cells with 5‐ethynyl‐2′‐deoxyuridine (EdU) and immature neurons with doublecortin (DCX) in the hippocampus (Figure 2F). Thus, EdU/DCX double‐positive cells were identified as proliferating neuronal progenitors. CIS reduced the number of EdU+ (Figure 2G), DCX+ (Figure 2H), and EdU/DCX double‐positive cells (Figure 2I), indicating impaired neurogenesis processes, including both proliferation and differentiation. However, the exercise intervention mitigated this reduction, thus demonstrating protective effects on neurogenesis under stressful conditions. Taken together, these results suggest that RPLP2 is a crucial exercise‐induced factor that facilitates hippocampal neurogenesis and contributes to anxiety resistance.

### 2.3 Hippocampal RPLP2 Ablation Induces Anxiety‐Like Behaviors That Can be Reversed by Exercise

To determine whether reduced hippocampal RPLP2 levels are a causal factor in anxiety and to define the functional role of the protein in exercise‐induced neurogenesis and anxiety resistance, we knocked down RPLP2 expression specifically in the dentate gyrus (DG), a key region for hippocampal neurogenesis [34], as global knockout of this gene is lethal. We performed this knockdown through stereotaxic injections of an adeno‐associated virus (AAV) encoding short‐hairpin RNA targeting RPLP2 (AAV‐shRPLP2) (Figure 3A). To verify the anatomical specificity of the RPLP2 knockdown, brain sections from virus‐injected mice were stained with an RPLP2 antibody; subsequent analyses revealed that the viral signal was localized mainly within the dentate gyrus. Consistent with these findings, RPLP2 immunoreactivity was reduced predominantly in the DG region, whereas no obvious reductions were observed in adjacent hippocampal regions, supporting a relatively DG‐specific knockdown of RPLP2 (Figure S4A). These mice were then stratified into sedentary and voluntary exercise groups; note that neither viral injection nor the exercise intervention affected basic physiological parameters such as locomotor activity (Figure S4B) or body weight (Figure S4C). Moreover, exercise alone did not alter hippocampal RPLP2 transcription (Figure S4D). The results of quantitative polymerase chain reaction (qPCR) confirmed the successful reduction in RPLP2 messenger RNA (mRNA) expression in the DG of RPLP2‐knockdown mice; moreover, in this background, exercise did not restore hippocampal RPLP2 mRNA levels (Figure 3B). At the protein level, however, exercise rescued hippocampal RPLP2 protein expression (Figure 3C). DG RPLP2 knockdown markedly impaired neurogenesis (Figure 3D–G) and promoted anxiety‐like behaviors (Figure 3H,I) without affecting locomotion (Figure S4E). Notably, exercise fully rescued both neurogenesis and behavioral deficits in RPLP2‐knockdown mice, restoring them to the levels seen in control mice (Figure 3D–I). These results suggest that RPLP2 contributes to the maintenance of neurogenesis and normal affective behavior, and indicate that exercise can compensate for the loss of its protein levels within the hippocampus. Given that circulating RPLP2 levels are increased by exercise (Figure 2D) and that hippocampal RPLP2 mRNA levels are not correlated with hippocampal RPLP2 protein levels, we propose that the increase in hippocampal RPLP2 levels caused by exercise is mediated primarily by an influx from peripheral tissues.

**FIGURE 3:**
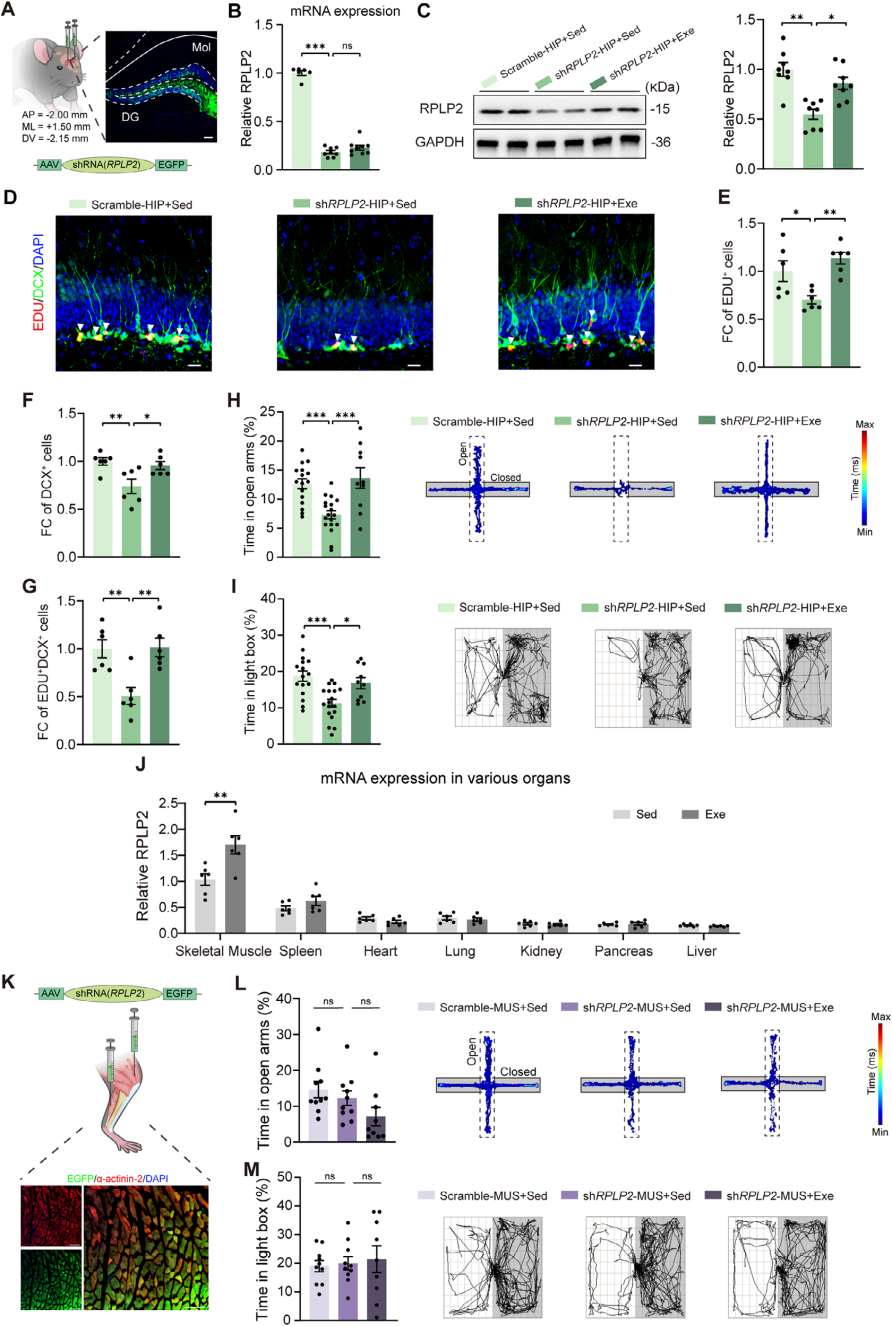
Voluntary running mitigates impaired neurogenesis and anxiety‐like behaviors in mice with ablated hippocampal RPLP2. (A) pAAV‐U6‐shRNA(RPLP2)‐CMV‐EGFP (enhanced green fluorescent protein) was utilized for specific RPLP2 knockdown in the DG, with a representative image showing EGFP expression (on the right). Scale bars = 100 µm. (B) RPLP2 expression in the hippocampus as quantified via qPCR (*n* = 6‒9 mice per group). (C) Representative immunoblotting bands of RPLP2 from hippocampal tissue (left) and relative protein levels (right) (*n* = 8 mice per group). (D) Representative immunofluorescence images of hippocampal slices stained for EdU and DCX. White arrows indicate EdU/DCX double‐positive cells. Scale bar = 25 µm. (E–G) Fold change (FC) of (E) EdU+, (F) DCX+, (G) EdU+ and DCX+ cells (*n* = 6 mice per group). (H) Results of the EPM test, including proportion of time spent in the open arms of the maze and representative heatmaps of mouse movement trajectories (*n* = 10‒18 mice per group). (I) Results of the light/dark box test, including proportion of time spent in the light box and representative mouse movement traces (*n* = 10‒18 mice per group). (J) RPLP2 gene expression in multiple organs of mice as quantified via qPCR (*n* = 6 mice per group). (K) Schematic illustration of pAAV‐U6‐shRNA(RPLP2)‐CMV‐EGFP virus injection into the hindlimb muscles of the mice and representative immunofluorescence images showing viral infection in muscle tissue alongside α‐actinin‐2 staining. Scale bar = 250 µm. (L) Results of the EPM test, including proportion of time spent in the open arms of the maze and representative heatmaps of mouse movement trajectories (*n* = 9‒10 mice per group). (M) Results of the light/dark box test, including proportion of time spent in the light box and representative mouse movement traces (*n* = 9‒10 mice per group). Sed: sedentary, Exe: exercise, HIP: hippocampus, MUS: muscle. Statistical analyses, including two‐tailed unpaired *t*‐tests, one‐way ANOVA with Tukey's posthoc test, Welch's ANOVA with Games–Howell posthoc test, and Kruskal–Wallis test with Dunn's posthoc test, were performed using GraphPad Prism 9. All values are presented as the mean ± SEM; ns: not significant; *p < 0.05, **p < 0.01, and ***p < 0.001.

To determine the primary source of exercise‐induced, peripheral‐circulating RPLP2, we first investigated the expression pattern of RPLP2 mRNA across multiple organs and found that skeletal muscle exhibited the highest expression under baseline conditions (Figure 3J). After 28 days of voluntary exercise, RPLP2 expression was significantly upregulated specifically in muscle, and no changes were detected in other tissues (Figure 3J). We then measured RPLP2 levels in mice subjected to hippocampal RPLP2 knockdown and found that exercise increased both muscular (Figure S4F) and circulating (Figure S4G–H) RPLP2 protein levels, indicating that these changes occurred independently of hippocampal RPLP2 expression. Collectively, these findings indicate that skeletal muscle is likely an important source of exercise‐associated circulating RPLP2. Circulating RPLP2 derived from muscle may compensate for the reduction in the hippocampal levels of the protein, thereby mediating the beneficial effects of exercise on neurogenesis and anxiety.

We subsequently aimed to investigate whether the specific RPLP2 silencing in muscle tissue affects stress resistance in mice. Given that global RPLP2 knockout is lethal in mice, we adopted an AAV–shRNA‐mediated knockdown strategy. Owing to the key locomotory function of the hindlimb muscles, which represent a substantial proportion of the total muscle mass of the body [35, 36] and their well‐documented metabolic sensitivity to exercise [37, 38], the virus was locally injected into these muscles (Figure 3K and Figure S5A). After a 3‐day recovery, all the mice were subjected to an exercise intervention. Neither running performance nor body weight gain differed significantly between the groups (Figure S5B,C). Effective RPLP2 knockdown in the skeletal muscle, confirmed at both the mRNA and protein levels, was not rescued by the exercise intervention (Figure S5D,E). Moreover, no off‐target transcriptional changes were identified in other organs (Figure S5F), indicating that the viral‐mediated knockdown was muscle specific. Unexpectedly, muscular RPLP2 ablation did not induce anxiety‐like behaviors (Figure 3L,M and Figure S5G) or affect hippocampal neurogenesis (Figure S5H–K). Additionally, both the mRNA expression and protein levels of hippocampal RPLP2 were unchanged by the skeletal muscle–targeted knockdown, regardless of exercise status (Figure S5L,M). Therefore, we propose that under physiological conditions, changes in muscle RPLP2 levels are unlikely to result in substantial alterations in stress resilience if the hippocampal RPLP2 levels are not also altered. These findings suggest that under physiological conditions, muscle‐derived RPLP2 may not be strictly indispensable; instead, compensatory input from other sources and/or parallel mechanisms may partially protect hippocampal neurogenesis and support behavioral regulation. In contrast, in experimental paradigms involving pre‐existing vulnerability factors such as CIS or targeted hippocampal RPLP2 knockdown, the exercise‐driven elevations in peripheral circulating RPLP2 levels may reflect contributions from muscles and contribute to counteracting local hippocampal protein deficits and attenuating anxiety‐like behaviors.

### 2.4 Muscular RPLP2 Is Important for Transferring Exercise‐Mediated Anxiolysis in Mice With Ablated Hippocampal RPLP2

Next, we aimed to determine whether exercise‐driven neurogenesis and anxiolysis are channeled through a muscle–brain axis in hippocampal RPLP2‐deleted mice. To address this aim, we designed a dual‐site knockdown experiment specifically targeting RPLP2 in both the hindlimb muscles and the hippocampus of mice and subsequently allowed the mice to engage in voluntary exercise (Figure 4A). Following knockdown, the mice presented normal weight (Figure S6B) and locomotor activity (Figure S6A,C). Immunoblotting analysis subsequently confirmed the successful RPLP2 knockdown in both muscles and hippocampal tissues (Figure 4B,C). Notably, RPLP2 expression ablation in the hindlimb muscles completely mitigated the exercise‐induced increase in RPLP2 levels in the hippocampus (Figure 4C). Immunofluorescence staining revealed that the exercise intervention failed to rescue weak neurogenesis in mice in whom RPLP2 in the hippocampus, and muscle was concurrently silenced, leading to a significant decrease in the number of newborn neurons (Figure 4D–G). Behavioral assessments further demonstrated that the blocking of muscle‐derived RPLP2 expression significantly counteracted the running‐induced anxiolytic effects in stressed mice (Figure 4H,I). These findings strongly underscore the role of muscle‐derived RPLP2 in modulating hippocampal neurogenesis and anxiety‐like behaviors.

**FIGURE 4:**
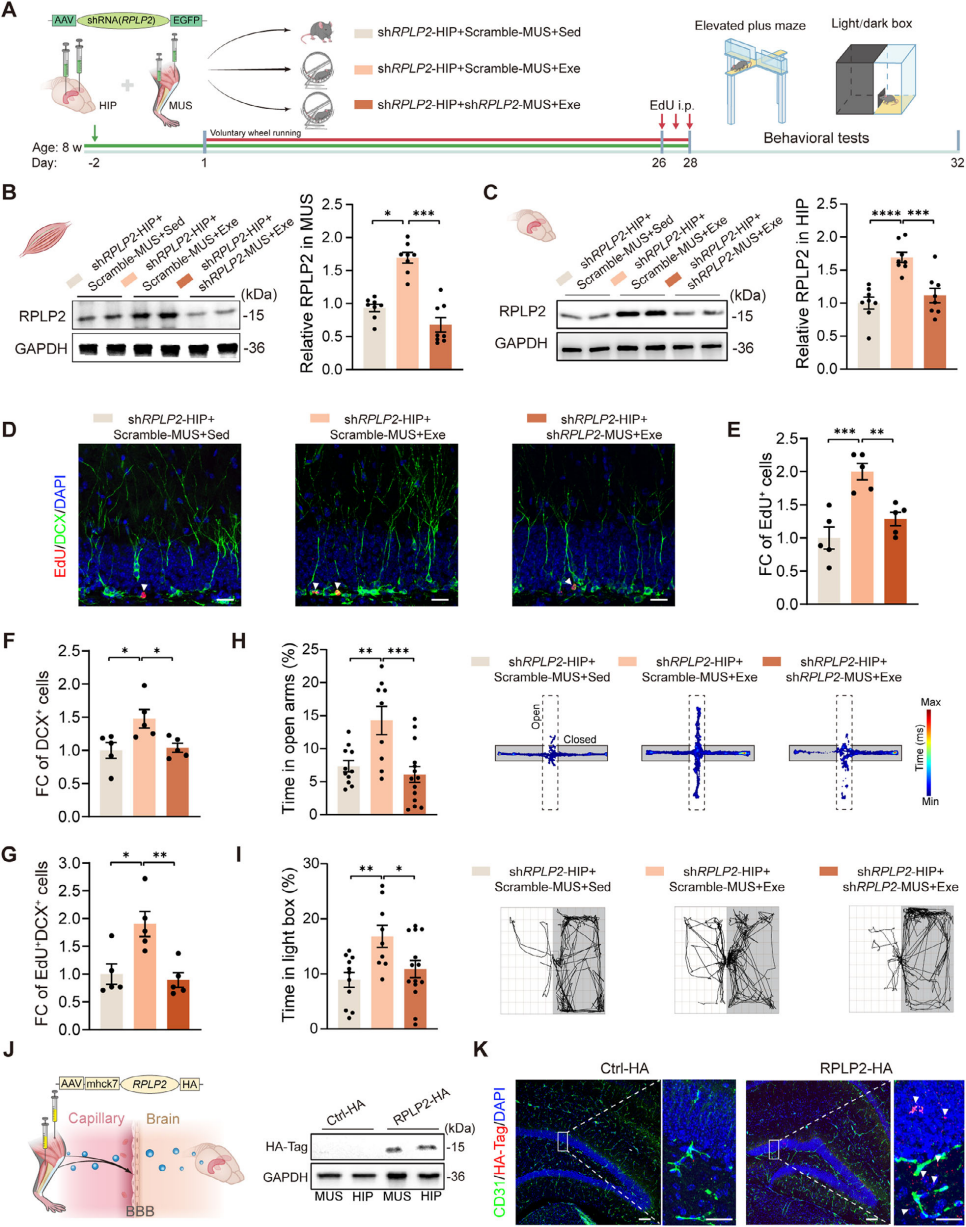
Silencing of skeletal muscle RPLP2 expression attenuates the beneficial effects of running on neurogenesis and stress resilience in mice with ablated hippocampal RPLP2. (A) Overview of the study design, including pAAV‐U6‐shRNA(RPLP2)‐CMV‐EGFP virus injection into the muscles and the hippocampus, establishment of a voluntary running wheel exercise model, and several behavioral testing procedures. (B) Representative immunoblotting bands of RPLP2 from muscle tissue and relative protein levels (*n* = 8 mice per group). (C) Representative immunoblotting bands of RPLP2 from hippocampal tissue and relative protein levels (*n* = 8 mice per group). (D) Representative immunofluorescence images of hippocampal slices stained for EdU and DCX. White arrows indicate EdU/DCX double‐positive cells. Scale bar = 20 µm. (E–G) Fold change (FC) of (E) EdU+, (F) DCX+, and (G) EdU/DCX double‐positive cells (*n* = 5 mice per group). (H) Results of the EPM test, including proportion of time spent in the open arms of the maze and representative heatmaps of mouse movement trajectories (*n* = 9‒14 mice per group). (I) Results of the light/dark box test, including proportion of time spent in the light box and representative mouse movement traces (*n* = 9‒14 mice per group). (J) Administration of SCAAV‐mhck7‐HA‐WPRES for specific overexpression of HA‐tagged RPLP2 in the muscles (left), and representative immunoblotting bands of RPLP2‐HA tags from hippocampal and muscle tissues (right). (K) Representative immunofluorescence images of hippocampal slices stained for CD31 and HA tag. Scale bar = 100 µm (left) and 25 µm (right). Sed: sedentary, Exe: exercise, Ctrl: control, MUS: muscle, HIP: hippocampus. Statistical analyses, including one‐way ANOVA with Tukey's posthoc test and Kruskal–Wallis test with Dunn's posthoc test, were performed using GraphPad Prism 9. All values are presented as the mean ± SEM; *p < 0.05, **p < 0.01, ***p < 0.001, and ****p < 0.0001.

To investigate whether muscle‐derived RPLP2 can reach the hippocampus, we injected a viral vector into the mouse hindlimb muscles, as these are the muscles that are predominantly engaged during exercise. The vector was designed to express hemagglutinin (HA)‐tagged RPLP2 under the control of the muscle‐specific promoter mhck7, ensuring protein expression in muscle cells. To validate this muscle RPLP2‐overexpression model, we measured HA expression in the serum and major peripheral tissues via western blotting. HA signals were readily detected in the injected skeletal muscle (Figure 4J), confirming effective local overexpression, whereas no such signals were evident in the examined nonmuscular organs (Figure S6D), supporting the predominant restriction of viral expression to skeletal muscle. HA signals were also detected in serum, indicating that muscle‐overexpressed HA‐RPLP2 entered the circulation (Figure S6E). In addition, immunoblotting analysis of hippocampal tissue revealed the presence of HA‐tagged RPLP2 (Figure 4J); compared with those in animals treated with the control viral vector, HA‐tagged RPLP2 was markedly enriched in the hippocampus and DCX+ cells in virus‐treated animals (Figure 4K and Figure S6F). Taken together, these results confirm the efficiency and tissue specificity of the muscle overexpression approach and support the interpretation that muscle‐derived RPLP2 can enter the circulation and thus be delivered to the hippocampus. Collectively, our findings identify RPLP2 as an exercise‐driven, muscle‐derived circulating factor that contributes to facilitating neurogenesis and alleviating anxiety‐like behaviors in model mice. We further delineated a muscle–brain axis through which muscle‐derived RPLP2 mediates the beneficial effects of exercise, including the restoration of stress resilience under anxiety‐like conditions.

### 2.5 RPLP2 Promotes Ribosomal Localization and Dynamic Assembly in NSPCs

To elucidate the mechanisms through which RPLP2 influences neurogenesis, we conducted a comprehensive series of in vitro cellular experiments. Neural stem or progenitor cells (NSPCs) were isolated, cultured, and exposed to supernatant from the culture medium of myoblast cell line C2C12, a mouse skeletal muscle cell line widely used for studying differentiation and regeneration (Figure 5A). To validate the role of RPLP2 in neurogenesis, we immunodepleted RPLP2 from muscle cell‐conditioned medium (MCCM) using magnetic beads (Figure 5A). RPLP2‐depleted MCCM‐cultured NSPCs exhibited reduced proliferation and differentiation compared with those cultured with full MCCM (Figures S7A and 5B).

**FIGURE 5:**
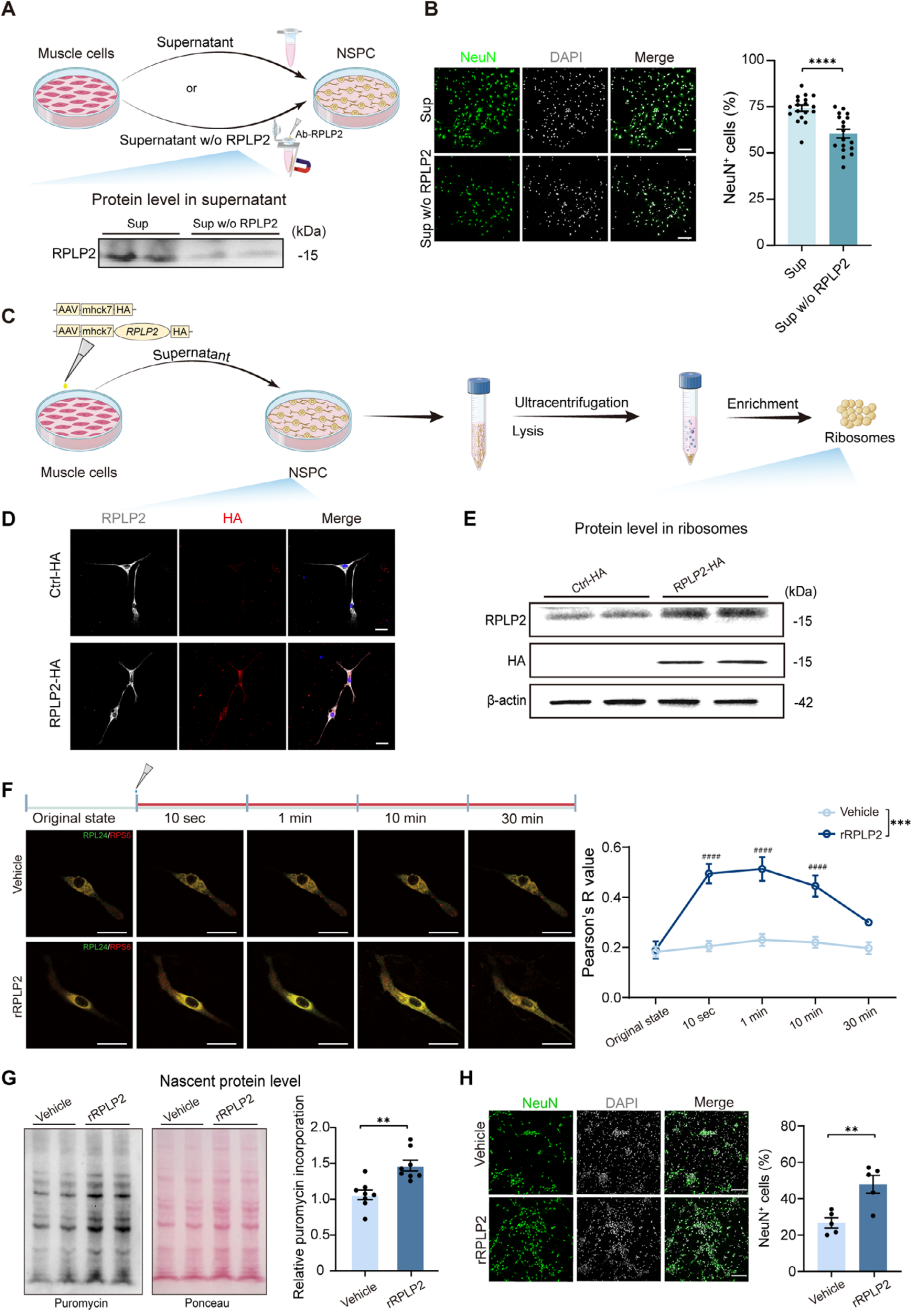
Muscle cell–derived RPLP2 is required for ribosomal localization and rapid assembly in NSPCs. (A) Schematic of the generation of muscle cell–conditioned media, whose supernatant was used for culturing NSPCs. Representative western blotting bands of RPLP2 from the supernatant of muscle cell culture medium. (B) Representative immunofluorescence staining of differentiated NSPCs cocultured with MCCM under different treatment conditions for NeuN expression (left) and NeuN+ cell counts (right) (*n* = 3 independent experiments with 6 biological replicates each). Scale bar = 100 µm. (C) Schematic representation of conditional NSPC culture followed by ultracentrifugation for isolating ribosomes. (D) Representative immunofluorescence staining for RPLP2 and HA in differentiated NSPCs following culture with conditioned medium from C2C12 cells transduced with either HA or RPLP2‐HA. Scale bar = 25 µm. (E) Representative immunoblotting bands of HA and RPLP2 from NSPC ribosomes. (F) Representative live imaging of RPL24 (green) and RPS6 (red) before and 10 s, 1 min, 10 min, and 30 min after rRPLP2 stimulation (left). The colocalization tendency of RPL24 and RPS6 signals was estimated by Pearson's *R* values (about threshold) shown on the right (*n* = 2 independent experiments with 3 biological replicates each). Scale bar = 25 µm. (G) Relative nascent protein levels (right) and representative immunoblotting bands of nascent proteins from NSPCs upon differentiation after culture with vehicle or rRPLP2 via the incorporation of puromycin (left). (H) NeuN+ cell counts (right) and representative immunofluorescence staining of NeuN upon differentiation in cells cultured with vehicle or rRPLP2 (left) (*n* = 5 independent experiments per group). Scale bar = 100 µm. NSPC: neural stem or progenitor cell, Ab‐RPLP2: RPLP2 antibody for immunoprecipitation, Sup: supernatant, w/o: without. Statistical analyses, including two‐tailed unpaired *t*‐tests, one‐way ANOVA with Tukey's posthoc test, and repeated‐measures ANOVA followed by Bonferroni multiple comparisons test, were performed using GraphPad Prism 9. All values are presented as the mean ± SEM; *: Significant overall main effect between groups; #: rRPLP2 versus vehicle at matched time points. **p < 0.01, ***p < 0.001, ****p < 0.0001, and ####*p* < 0.0001.

Given the important role of RPLP2 as a ribosomal protein integral to protein synthesis, we subsequently investigated whether RPLP2 promotes ribosome function through direct integration into neuronal ribosomes. To this end, we generated MCCM containing HA‐tagged RPLP2 secreted by C2C12 cells transduced with an AAV‐mhck7‐RPLP2‐HA vector. We then treated NSPCs with this MCCM; subsequent immunofluorescence staining confirmed the presence of HA‐tagged RPLP2 in the NSPCs (Figure 5C(left panel),D). Ribosomes were subsequently isolated from the NSPCs via ultracentrifugation, and subsequent immunoblotting analysis revealed the presence of HA in the ribosomal fraction of the NSPCs (Figure 5C(right panel),E). Conversely, upon treatment with MCCM derived from muscle cells infected with a control virus, neither the NSPC nor the ribosomal fraction exhibited HA. These results suggest that muscle‐derived RPLP2 is directly incorporated into NSPC ribosomes.

To investigate how RPLP2 enters cells, we constructed an exogenous recombinant RPLP2 (rRPLP2) (Figure S7B). With this construct, we first investigated its ability to cross the blood–brain barrier (BBB). To this end, Cy5.5‐conjugated rRPLP2 protein was administered to mice via tail vein injections (Figure S7C). Subsequent immunofluorescence analysis of brain sections revealed robust Cy5.5 signals within the DG (Figure S7D), suggesting that peripherally administered rRPLP2 can reach the targeted brain area. To further define the route by which rRPLP2 enters hippocampal neural stem cells, we co‐stained the cells for the early endosome marker EEA1, the lysosomal marker LAMP1, and the neural stem cell marker Nestin. Histological analysis revealed that Cy5.5 colocalized with EEA1 (Figure S7E), suggesting that it was taken up via endocytosis. Some of the Cy5.5 signal also overlapped with that of LAMP1 (Figure S7F), indicating that a portion of internalized rRPLP2 is taken up by lysosomes for degradation, whereas the remaining protein may be released into the cytoplasm, allowing exertion of its function. These results suggest that RPLP2 enters hippocampal neural stem cells through endocytosis to mediate its effects.

Next, we examined the functional effects of RPLP2 on ribosomal dynamics. To visualize ribosome assembly in real time, NSPCs expressing fluorescently tagged large (RPL24‐EGFP) and small (RPS6‐mCherry) ribosomal subunits were treated with rRPLP2. Live‐cell imaging revealed a rapid and significant increase in the colocalization of RPL24 and RPS6 within 10 s of stimulation, indicating accelerated subunit association (Figure 5F). This enhanced colocalization was transient, however, and returned to baseline within 30 min. High‐resolution imaging of fixed NSPCs at defined time points post stimulation confirmed the marked increase in ribosomal subunit assembly at 10 s and 1 min (Figure S8A), which was consistent with the live‐cell data. Collectively, these results indicate that elevated RPLP2 increases the efficiency of ribosomal subunit assembly to support subsequent translation.

The spatial dynamics of ribosomes, reflecting RPLP2 function, were subsequently investigated through subcellular distribution analysis. We used ribosomal protein S6 (RPS6), a key component of the 40S ribosomal subunit, as a marker for ribosomal localization in NSPCs. Immunofluorescence colocalization analysis was conducted to assess the overlap between RPS6 (red fluorescence) and 4′,6‐diamidino‐2‐phenylindole (DAPI) (blue fluorescence) along the axis traversing the nucleolar center. In NSPCs treated with MCCM supernatant, the nuclei exhibited minimal RPS6 and DAPI signal colocalization; instead, red fluorescence was predominantly concentrated in MAP2‐positive dendritic regions (green fluorescence) (Figure S8B). In contrast, RPS6 was localized primarily to the nucleus and perinuclear cytoplasm in the RPLP2‐depleted MCCM groups, with limited dendritic residence (Figure S8B). In eukaryotic cells, ribosomes are composed of a large 60S subunit and a small 40S subunit [39], which assemble in the nucleolus and are subsequently transported to the cytoplasm through the nuclear pore complex to initiate their translation functions [40]. Ribosomes, which are widely detected in neuronal dendrites and axons and are capable of local translation, play crucial roles in synaptic protein production and repair, explaining the importance of their redistribution to the dendrites of neurons [23]. The unexpected alteration in ribosomal distribution observed in NSPCs under rRPLP2 or MCCM exposure suggests a role for RPLP2 in promoting ribosomal redistribution from the nucleus to the cytoplasm and ultimately to the dendrites. This redistribution may facilitate local protein synthesis, which is crucial for neuronal proliferation and differentiation. To verify this, we used puromycin to label nascent proteins and found that rRPLP2 significantly increased nascent protein synthesis (Figure 5G). In addition, the levels of rRPLP2 recapitulated the enhanced NSPC proliferation and differentiation observed with MCCM treatment (Figure S8C and Figure 5H). Overall, these findings indicate that RPLP2 rapidly promotes the transient assembly of large and small ribosomal subunits in NSPCs and facilitates ribosomal redistribution from the nuclear/perinuclear region to the cytoplasm and dendrites, accompanied by increased nascent protein synthesis and enhanced NSPC proliferation and differentiation.

In summary, our findings indicate that muscle cell–derived RPLP2 stimulates NSPC proliferation and differentiation by regulating ribosome localization and dynamics. Specifically, RPLP2 is incorporated into the ribosomes of NSPCs, amplifies the association of ribosomal subunits, and facilitates the redistribution of ribosomes to the dendrites, thereby sustaining local protein synthesis, which is essential for neuronal regeneration.

### 2.6 RPLP2 Is Required for Transcriptional Landscape Underlying NSPC Proliferation and Neuronal Development

To elucidate the molecular mechanisms underlying RPLP2‐mediated neurogenesis and anxiolysis, we conducted a comprehensive transcriptomic analysis of NSPCs treated with AAV–shRPLP2, AAV–shRPLP2 supplemented with rRPLP2, or a scrambled AAV control (Figure 6A) via RNA sequencing (RNA‐seq). Compared with the control group, the shRPLP2 group exhibited a notable downregulation of 264 genes, whereas 953 genes were markedly upregulated in the shRPLP2 + rRPLP2 group compared with the shRPLP2 group (Figure 6B,C). Within this dataset, we identified 108 genes that were bidirectionally regulated upon RPLP2 silencing and subsequent restoration (Figure 6D). Gene Ontology (GO) enrichment analysis revealed that these 108 genes were significantly enriched in three functional categories: ion binding, extracellular matrix (ECM), and transcriptional regulation. These findings suggested that RPLP2 may act as an upstream regulatory factor, coordinating ion‐mediated signals and ECM interactions to activate transcriptional programs that promote NSPC‐to‐neuron differentiation, as well as the formation and maturation of axons and dendrites (Figure 6E). Kyoto Encyclopedia of Genes and Genomes (KEGG) pathway analysis of these 108 genes revealed significant enrichment in pathways associated with cellular development and synaptic plasticity (Figure 6F).

**FIGURE 6:**
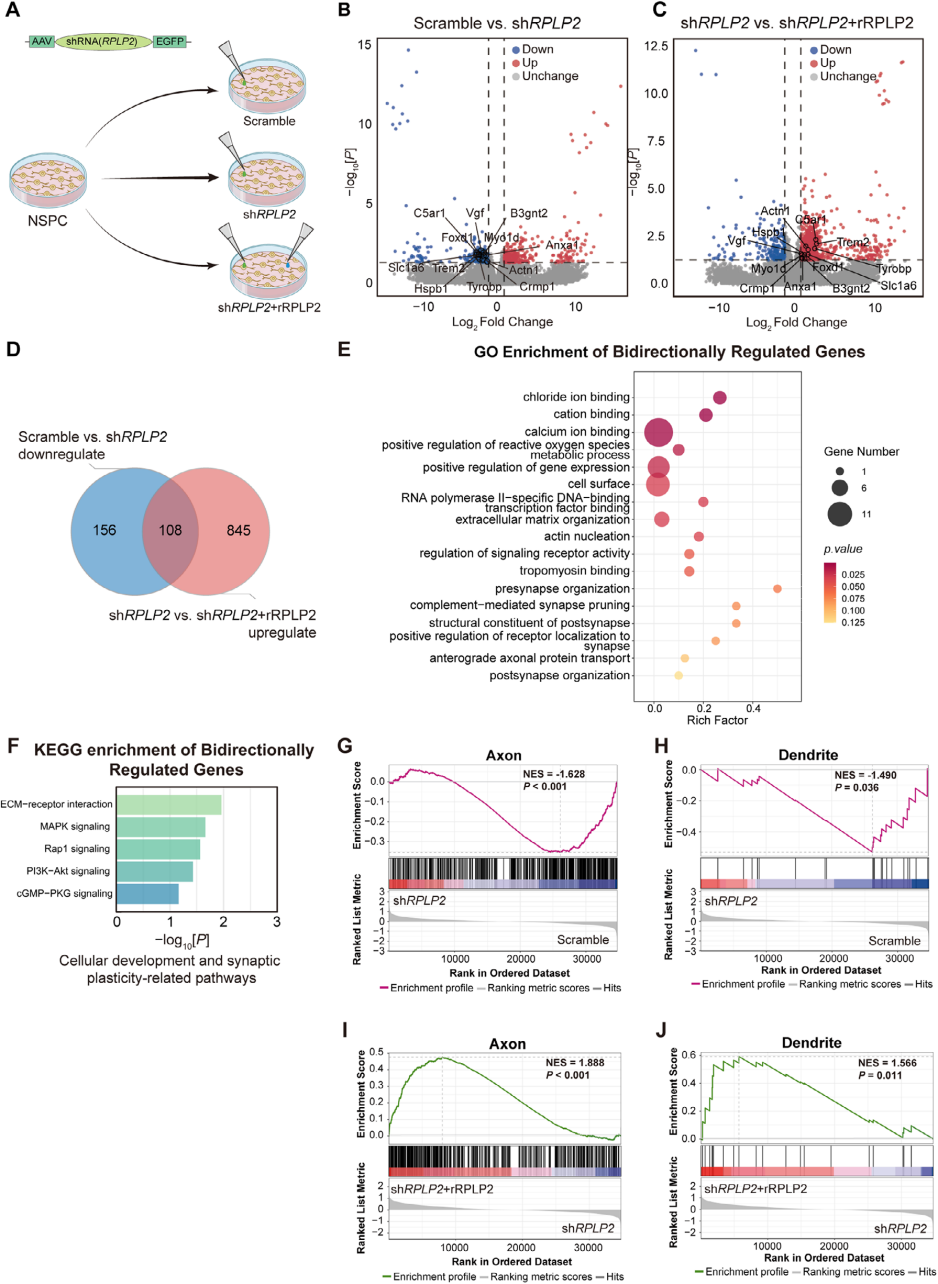
RPLP2 is involved in regulating a gene network crucial for synaptic development. (A) Schematic of the NSPC intervention protocol. NSPCs were subjected to RPLP2 knockdown via administration of pAAV‐U6‐shRNA(RPLP2)‐CMV‐EGFP and supplemented with exogenous rRPLP2. (B,C) Volcano plots displaying the DEPs (B) between the scramble and shRPLP2 groups and (C) between the shRPLP2 and shRPLP2 + rRPLP2 groups. (D) Venn diagram of differentially expressed genes. (E) GO analysis of 108 genes that were bidirectionally regulated upon RPLP2 silencing and subsequent restoration. (F) KEGG enrichment analysis identifying the pathways in which the bidirectionally regulated genes were enriched. (G–J) Gene set enrichment analysis results: (G,H) gene sets related to axons and dendrite development regulation exhibited significantly decreased normalized enrichment scores (NESs) after RPLP2 knockdown; (I,J) gene sets related to axons and dendrite development regulation showed significantly increased NESs after exogenous supplementation with rRPLP2.

We next performed a gene set enrichment analysis (GSEA), which revealed several pivotal targets associated with axon and dendrite development, including genes involved in cytoskeletal organization, synaptic formation, and neuronal signaling pathways. Notably, RPLP2 knockdown significantly impaired the enrichment of genes related to the regulation of axon and dendrite development (Figure 6G,H), whereas exogenous supplementation with rRPLP2 effectively restored this gene enrichment (Figure 6I,J), suggesting that RPLP2 plays a critical role in promoting neuronal development under these conditions. By integrating the results of the GO, KEGG, and GSEA pathway analyses, we conclude that RPLP2 not only regulates genes involved in cell proliferation and differentiation but also profoundly influences the expression of genes that are indispensable for neuronal morphology and function. Taken together, these findings emphasize the crucial role of RPLP2 in coordinating gene expression programs that are fundamental for neuronal development and synaptic plasticity.

### 2.7 rRPLP2 Treatment Recapitulates Exercise‐Induced Benefits by Enhancing Plasticity Among Newborn Neurons in Stressed Mice

To mimic the exercise‐induced increase in circulating RPLP2, we next evaluated whether peripheral injections of rRPLP2 could replicate the exercise‐induced benefits on synaptic plasticity, neurogenesis, and anxiolysis. Mice were first injected with an RV‐EF1α‐EGFP virus into the DG (Figure 7A), allowing the specific labeling of newborn neurons [41]. We then administered rRPLP2 to the mice via tail vein injections every other day for 28 days at a dose of 1.5 mg kg^−1^; at this dose, the virus produced comparable hippocampal RPLP2 levels after each injection to those induced by running (Figure S9A). From days 18 to 28, the mice were subjected to CIS daily for 2 h (Figure 7A). All mice exhibited normal weight (Figure S9B) and locomotor activity (Figure S9H). We subsequently evaluated whether systemic administration of rRPLP2 could restore hippocampal RPLP2 levels in mice subjected to CIS. Immunoblotting revealed that CIS markedly decreased hippocampal RPLP2 expression. Notably, compared with control treatment, rRPLP2 treatment increased hippocampal RPLP2 levels in the CIS group (Figure 7B), confirming the effectiveness of systemic rRPLP2 supplementation in restoring hippocampal RPLP2 levels.

**FIGURE 7:**
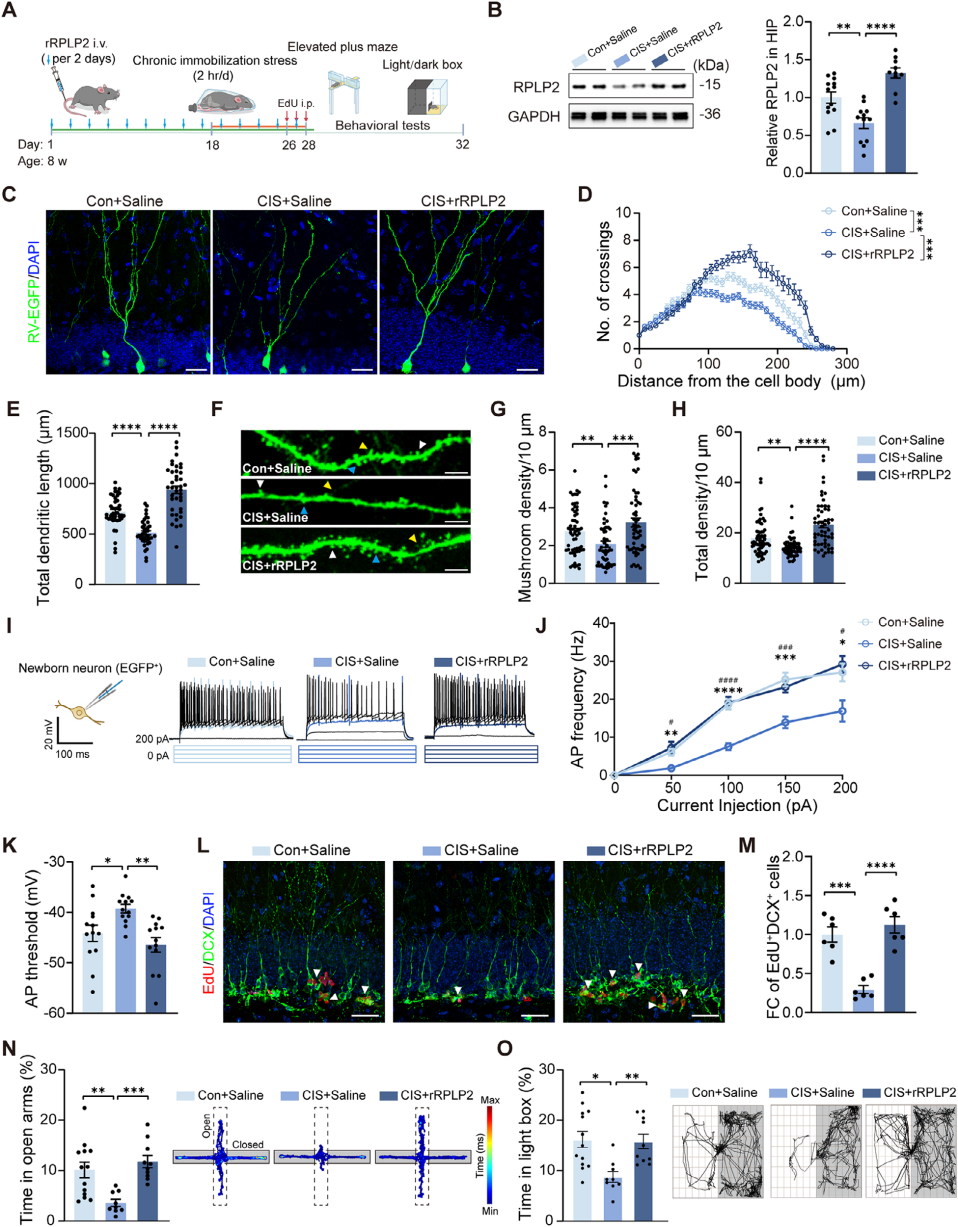
rRPLP2 administration mimics the beneficial effects of exercise by enhancing morphological maturation and plasticity among newborn hippocampal neurons in CIS mice. (A) Overview of the study design, including rRPLP2 administration via tail vein injections, CIS‐based induction of a mouse anxiety model, and behavioral testing. (B) Representative immunoblotting bands of RPLP2 from hippocampal tissue (left) and relative protein levels (right) (*n* = 9‒13 mice per group). (C–E) Representative (C) images, (D) dendritic complexity, and (E) total dendritic length of EGFP+ newborn neurons (*n* = 40 cells). Scale bars = 25 µm. (F–H) Representative (F) images and (G) mushroom‐shaped spine, and (H) total spine density of EGFP+ newborn neurons (*n* = 54–58 segments). White arrows indicate mushroom‐shaped spines; yellow arrows indicate long thin spines; and blue arrows indicate stubby spines. Scale bars = 5 µm. (I) Exemplary spike pattern of EGFP+ newborn neurons following the injection of current ranging from 0 to 200 pA. (J,K) Quantitative assessment of the (J) action potential firing frequency following rRPLP2 injection and (K) the threshold at which the first action potential was elicited (*n* = 13 cells). *: Significant difference between Con + Saline and CIS + Saline; #: Significant difference between CIS + Saline and CIS + rRPLP2. (L) Representative immunofluorescence images of hippocampal slices stained for EdU and DCX. White arrows indicate EdU/DCX double‐positive cells. Scale bar = 25 µm. (M) Fold change (FC) of EdU+ and DCX+ cells (*n* = 6 mice per group). (N) Results of the EPM test, including proportion of time spent in the open arms and representative heatmaps of mouse movement trajectories (*n* = 9‒13 mice per group). (O) Results of the light/dark box test, including proportion of time spent in the light box and representative mouse movement traces (*n* = 9‒13 mice per group). Con: control; CIS: chronic immobilization stress. Statistical analyses, including one‐way ANOVA with Tukey's posthoc test, repeated‐measures ANOVA, Welch's ANOVA with Dunnett's T3 posthoc test, and Kruskal–Wallis test with the Dunn's posthoc test, were performed using GraphPad Prism 9. All values are presented as the mean ± SEM; *p < 0.05, **p < 0.01, ***p < 0.001, ****p < 0.0001, #*p* < 0.05, ###*p* < 0.001, and ####*p* < 0.0001.

We next examined the morphology of the EGFP‐labeled newborn neurons within the DG (Figure 7C). Our findings revealed that CIS exposure markedly decreased the dendritic complexity and lengths of these neurons (Figure 7D,E). Notably, treatment with rRPLP2 mitigated these negative effects, as indicated by significant increases in dendritic branching and length in the CIS + rRPLP2 group compared with those in the CIS group (Figure 7D,E). To further assess synaptic plasticity, we comprehensively analyzed dendritic spine density and morphology (Figure 7F). Our results suggested that CIS exposure led to a substantial reduction in the number of mushroom‐shaped spines, which are indicative of mature synapses, as well as a decrease in the total spine count (Figure 7G,H). However, the number of long thin and stubby spines, which represent immature synaptic structures, remained unaffected by CIS exposure (Figure S9C,D). Intriguingly, the administration of rRPLP2 to CIS‐exposed mice resulted in significant increases in both the number of mushroom‐shaped spines and of total spines in the CIS + rRPLP2 group (Figure 7G,H). These observations suggest that rRPLP2 supplementation both preserves and increases synaptic connectivity and stability, potentially contributing to improved neuronal function and resilience.

Next, we performed electrophysiological recordings of EGFP‐labeled newborn neurons within the DG to assess their excitability (Figure 7I). Our results suggested that CIS exposure led to a significant decrease in action potential (AP) frequency (Figure 7J) and an increase in the AP threshold (Figure 7K), whereas the resting membrane potential remained unchanged (Figure S9E). Notably, treatment with rRPLP2 in the CIS + rRPLP2 group restored the AP frequency and threshold to levels comparable to those observed in control mice (Figure 7J,K). These findings support the ability of rRPLP2 to rescue neuronal excitability in mice exposed to stress.

Finally, we assessed the effects of rRPLP2 on neurogenesis and anxiety‐like behaviors in CIS mice. Our findings revealed that rRPLP2 robustly increased the numbers of EdU+, DCX+, and Edu/DCX double‐positive newborn neurons under both basal and stressful conditions (Figure 7L,M and Figure S9F,G), demonstrating its potent neurogenic properties. Furthermore, in the EPM and light/dark box tests, rRPLP2 strongly alleviated the anxiety‐like behaviors induced by CIS, restoring the animals’ performance to the levels similar to those in the control group (Figure 7N,O). These results suggest that the administration of rRPLP2 reverses stress‐induced impairments in neurogenesis and ameliorates anxiety‐like behaviors, thereby highlighting its potential to yield the neuroprotective benefits similar to those of physical exercise.

Taken together, these results indicate that the anxiolytic effects of rRPLP2 administration stem from its ability to rejuvenate neurogenesis and optimize the structure and function of newborn neurons. By fostering neural progenitor cell proliferation and differentiation, rRPLP2 administration replenishes the newborn neuron population, which is a crucial factor for sustaining hippocampal plasticity under stressful conditions. Concurrently, rRPLP2 treatment increases the structural intricacy, synaptic connectivity, and excitability of these neurons, thereby facilitating their integration into neural circuits. This dual mechanism not only restores hippocampal function but also improves the resilience of neural networks against stress‐induced insults. Collectively, our results show that exercise‐induced muscle RPLP2 promotes ribosomal assembly in newborn hippocampal neurons, thereby restoring neurogenesis and ultimately alleviating anxiety‐like behaviors.

## 3 Discussion and Limitations of the Study

Physical exercise exerts well‐known beneficial effects on mental health, notably the mitigation of anxiety and increased stress resistance. Recent evidence indicates that several exercise‐induced factors, such as brain‐derived neurotrophic factor (BDNF) [42], selenium [43], and clusterin [13], emulate the neurogenic effects associated with exercise. In addition, exercise has been shown to alter cortical RNA methylation [44] and synaptic protein lactylation [45], processes that potentiate neuroplasticity and contribute to stress resistance. On the basis of this growing evidence base, our study offers perspectives on the function of peripheral RPLP2, a newly identified myokine, in exercise‐induced anxiolysis and elucidates a previously unexamined muscle–brain axis.

Previous studies have demonstrated that compounds elicited by exercise, including myokines such as irisin [46], cathepsin B [47], and Interleukin‐6 (IL‐6) [18], as well as hepatokines such as Fibroblast Growth Factor‐21 (FGF‐21) [48], Insulin‐like Growth Factor‐1 (IGF‐1) [49], and Gpld1 [11], facilitate intricate communications between peripheral organs and the brain. This study further contributes to the field by including RPLP2 among existing exercise‐induced peripheral signaling molecules and supporting its classification as a novel myokine. Importantly, unlike canonically secreted cytokines or growth factors, RPLP2 is closely associated with ribosomal function. Therefore, the importance of our findings lies not only in the identification of a relatively novel exercise‐responsive factor but also in the implication that exercise‐induced peripheral signals, in addition to traditional trophic, inflammatory, and metabolic functions, may be involved in translational regulation. Conceptually, these findings broaden our understanding of the molecular modalities through which peripheral exercise signals communicate with the brain.

We found that RPLP2 knockdown in the hindlimb muscle completely abolished the exercise‐induced elevation of RPLP2 in the hippocampus and abolished the ability of exercise to restore hippocampal neurogenesis in chronically stressed mice. These findings indicate that exercise‐induced, muscle‐originating RPLP2 is not merely an accompanying change to exercise but rather an important effector molecule that actively mediates hippocampal plasticity remodeling and behavioral improvement. Together with the observation that exogenous RPLP2 supplementation can, to some extent, mimic the anxiolytic and proneurogenic effects of exercise, our results further support the importance of RPLP2 in linking skeletal muscle activity to adaptive brain responses. Future work should clarify how RPLP2 interacts with BDNF, IGF‐1, clusterin, and inflammation‐ or metabolism‐related signals to define its hierarchical position within the broader exercise‐responsive signaling network.

The hippocampus is a key brain region that regulates anxiety‐related behavior [50, 51]. Impaired hippocampal neurogenesis following chronic stress contributes significantly to the pathogenesis of anxiety disorders, a notion supported by the results of interventions that facilitate hippocampal neurogenesis, such as exercise, which promote stress resilience [52] and alleviate anxiety‐like behaviors [53, 54]. Consistent with these findings, our study revealed that CIS disrupted DG neurogenesis in close association with anxiety‐like phenotypes, while restoring neurogenesis, either through exercise or exogenous RPLP2 treatment, reversed these behavioral deficits, reinforcing the role of neurogenesis as a critical cellular substrate for stress resistance.

Although the present study focused on hippocampal neurogenesis, anxiety phenotypes arise fundamentally from interactions within a distributed network comprising several brain areas, including the amygdala, prefrontal cortex (PFC), and hippocampus [55, 56, 57]. Collectively, these structures support contextual processing, threat evaluation, emotional regulation, and behavioral output. Therefore, the hippocampus is unlikely to be the only brain region that mediates the anxiolytic effects of RPLP2 but is more likely to be an important site of action. In the present study, the behavioral improvements observed following injections of RPLP2 coincided with improved hippocampal neurogenesis, suggesting that hippocampal plasticity is at least one important mechanism underlying its effects. However, we did not systematically evaluate the effects of RPLP2 on the amygdala, the PFC, or the activity of related neural circuits. Accordingly, the possibility that extra‐hippocampal regions also contribute to the observed behavioral improvement cannot be excluded. Future studies combining region‐specific interventions, neural circuit tracing, and functional imaging analyses will help to more comprehensively define the scope of action of RPLP2 within the anxiety‐related brain network.

Mechanistically, one of the most notable findings of this study is that RPLP2 may link peripheral signals related to exercise to central translational regulatory programs. Ribosomes, which serve as the central orchestrators of protein synthesis, are precisely regulated to accommodate the dynamic requirements of cellular processes [23]. Emerging evidence indicates that these ribosomal proteins are not only located in the soma but are also transported to dendrites and axons, where they facilitate local translation, thereby modulating synaptic plasticity and neuronal function [58, 59, 60]. Furthermore, ribosomal dynamics, including both assembly and disassembly, are increasingly recognized as pivotal regulators of neuronal protein synthesis [61]. The acidic ribosomal P protein family is known to play crucial roles in ribosome assembly and protein synthesis [20]. Among the members of this family, RPLP2 specifically dimerizes with RPLP1 and functions in conjunction with RPLP0 to form the ribosomal stalk, a structure that both facilitates translation factor recruitment and regulates the elongation phase of protein translation [21]. Previous studies on RPLP2 have largely focused on oncology, particularly investigating its role in glycolysis and proliferation in hepatocellular carcinoma [22]. The present study is the first to suggest that RPLP2 also has important biological functions in the brain.

RPLP2 not only stimulates hippocampal neurogenesis but also orchestrates the morphological and functional maturation of newborn neurons. Specifically, RPLP2 increases neurite complexity and length while improving multiple electrophysiological properties, thereby enabling newborn neurons to functionally integrate into hippocampal circuits. These finding observations that in neurons, RPLP2 not only underpins structural development but also drives functional maturation. Collectively, our data support a dual function of RPLP2 in coordinating neuronal maturation, highlighting its important contribution to exercise‐induced neuroplasticity and the integration of newly generated neurons into hippocampal networks.

Notably, the principal target cells of RPLP2 in the hippocampus have not yet been clearly defined. RPLP2 may act directly on neural stem or progenitor cells to promote proliferation and differentiation; alternatively, it may primarily target immature neurons, facilitating morphological and functional maturation. In addition, RPLP2 may also indirectly improve the neurogenic niche by modulating astrocytes, microglia, or the local vascular‐associated microenvironment. Particularly under chronic stress, the inflammatory status, glial activation, and changes in the local metabolic milieu may influence hippocampal neurogenesis. In line with this idea, one study shows that peripheral Neutrophil Extracellular Traps (NETs) permeate damaged BBB and release Lcn2 to drive astrogliosis and mood disturbances [62]. Therefore, whether RPLP2 exerts regulatory effects on the neurogenic niche warrants further in‐depth investigation. Elucidating its cell type–specific targets will help more precisely define its mechanisms of action and optimize subsequent intervention strategies.

Intriguingly, our data revealed that relative to healthy controls, individuals with anxiety disorders exhibit significantly lower levels of RPLP2, whereas engagement in exercise elevated circulating RPLP2 concentrations in both humans and mice. These observations suggest that RPLP2 could function as a circulating biomarker of anxiety resistance. Furthermore, the systemic administration of RPLP2 appeared to partially reproduce the anxiolytic actions of exercise, underscoring its promise as a therapeutic candidate for anxiety disorders. In the present study, mice were administered Cy5.5‐conjugated rRPLP2 protein through tail vein injections, resulting in the detection of robust Cy5.5 signals within the DG. These findings emphasize the remarkable capacity of RPLP2 to permeate the BBB, although the precise mechanisms responsible for this crossing remain elusive. In addition, although our results indicate that muscle‐derived RPLP2 was detected in both the circulation and the hippocampus, the biochemical form of circulating RPLP2 was not determined. RPLP2 may circulate as a free soluble protein, within extracellular vesicles such as exosomes, or in association with carrier protein complexes. These distinct circulating forms may influence its stability in blood, transport across the BBB, tissue targeting, and cellular uptake in different ways. The results of our staining experiments suggest that RPLP2 may enter target cells through an endocytosis‐related process; however, these data did not reveal whether the internalized RPLP2 originates as a free protein, vesicle‐associated RPLP2, or carrier‐bound RPLP2. Future studies involving serum fractionation, extracellular vesicle isolation, density‐gradient separation, protease protection assays, RPLP2‐containing complex immunoprecipitation, and endocytosis inhibition approaches will be needed to clarify the circulating form of RPLP2 and the mechanism by which it is delivered to hippocampal cells. Furthermore, given its molecular weight (approximately 15 kDa) [20], RPLP2 has emerged as an exemplary candidate for mimicking or providing therapeutic benefits similar to exercise [63]. Clarifying its state and delivery mechanism is therefore essential for understanding RPLP2‐mediated muscle–brain communication.

Although existing, large‐scale public exercise datasets, including MetaMEx [64] and MoTrPAC [65], which respectively provide curated meta‐analytic evidence of exercise‐responsive molecular signatures and multiomics profiling of molecular transducers of physical activity, offer valuable resources for understanding systemic molecular adaptations to exercise, RPLP2 does not appear to show consistent exercise‐responsive patterns across these resources. This observation is important, as it suggests that RPLP2 should not be viewed as a universal exercise‐regulated molecule across all tissues, species, or exercise paradigms. Importantly, the information contained in these datasets differs from that obtained in our study in terms of both the biological context and measurement modality, as they were not designed to evaluate circulating RPLP2 in the context of exercise‐induced stress resistance. Many available datasets primarily profile tissue transcriptomes or bulk tissue proteomes, whereas circulating protein abundance may be regulated through mechanisms including altered secretion, extracellular release, protein stability, or tissue‐to‐blood redistribution, processes that may not be directly reflected by tissue mRNA levels or total intracellular protein abundance. Several additional factors may also account for the discrepancy between our findings and those derived from these public datasets, including species, exercise modality and intensity, intervention duration, sampling time relative to the last exercise session, the physiological or stress‐related state of the subjects, and the analytical platform. In particular, our study examined RPLP2 in the context of chronic exercise‐associated stress adaptation, which may involve muscle–brain communication programs distinct from those observed in unstressed or metabolically distinct populations. Thus, the lack of a uniform RPLP2 signature between MetaMEx and MoTrPAC and our data does not necessarily contradict our findings but rather highlights the context‐dependent nature of exercise‐modulated circulating factors. Notably, exercise‐induced alterations in RPLP2 expression were consistently validated across independent experiments and cohorts via proteomic sequencing, real‐time PCR (RT–qPCR) and western blotting, all yielding statistically significant results. Taken together, our results from both human interventions and mouse stress‐resilience experiments suggest that RPLP2 is an exercise‐associated circulating factor.

In summary, our findings suggest that muscle‐derived RPLP2 mediates the anxiolytic effects of exercise by promoting ribosome assembly and neurogenesis in the hippocampus. These results clarify molecular pathways that drive exercise‐induced stress resilience, which again drive exercise‐induced stress resistance and imply that RPLP2 may be a promising candidate as a therapeutic strategy for anxiety disorders.

### 3.1 Limitations of the Study

While our study provides substantial evidence that RPLP2 participates in exercise‐associated neurogenesis and anxiety resistance, several critical limitations remain. First, the pharmacological properties of RPLP2 remain unclear. Specifically, key issues such as its in vivo half‐life, the duration of its exercise‐induced elevation, the time required for its levels to return to baseline, and relevant tissue distribution, clearance pathways, and blood–brain barrier transport kinetics warrant further investigation; addressing these questions will be essential for clarifying how RPLP2 functions and for evaluating its feasibility as a potential therapeutic agent. Second, the receptor or direct downstream signaling pathway through which extracellular RPLP2 acts on the brain remains unknown. Future studies should consider using ligand–receptor screening, affinity purification coupled with mass spectrometry, receptor‐blocking or knockdown approaches, and transcriptomic/phosphoproteomic profiling to identify the direct molecular targets and signaling cascades activated by RPLP2 in neural cells and to understand how muscle‐derived RPLP2 is translated into improved adult hippocampal neurogenesis and behavioral outcomes. Third, although we suggested that RPLP2 may serve as a critical mediator of exercise‐induced neurogenesis and anxiolysis, a comparison of its efficacy with that of first‐line drugs, such as SSRIs, is necessary. Fourth, whether other myokines participate in the RPLP2‐mediated amelioration of anxiety remains unknown. Finally, large‐scale longitudinal cohorts are required to establish the reliability of RPLP2 as a circulating biomarker of anxiety resistance and to evaluate the long‐term safety and therapeutic efficacy of RPLP2‐based therapeutic interventions in clinical settings, particularly for patients with anxiety and motor disorders.

## 4 Materials and Methods

### 4.1 Study Design and Participants in the RCT

The RCT was designed and implemented according to the Consolidated Standards of Reporting Trials (CONSORT) and the enrollment and follow‑up took place from March 16 through October 18, 2021. All participants provided written informed consent. The trial was approved by the Ethics Committee of the First Affiliated Hospital of Xi'an Jiaotong University, China (Approval ID: XJTU1AF2020LSK‐276) and is registered in the Chinese Clinical Trial Registry (ChiCTR2100052776). The study drew on 272 undergraduate and graduate student volunteers from seven academic institutions. The inclusion criterion was an age of 18–35 years. The exclusion criteria were the presence or history of cardiovascular or respiratory diseases, neurological and psychiatric diseases, metabolic diseases, physical disabilities, severe dysmenorrhea resulting in an inability to exercise for more than 5 days, color blindness, and participation in a regular exercise routine.

### 4.2 Randomization and Interventions

Eligible participants were randomly and evenly assigned to either the exercise group or the control group via a computer‐generated randomization sequence. To maintain blinding, neither the data assessors nor the statisticians were informed of the group assignments. The 8‑week intervention protocol required the exercise group to complete regular running exercises (≥30 min per session, ≥3 times per week) [66, 67]. No additional intervention beyond self‑reported daily activities was provided to the control‑group participants. Keep software (Shenzhen, China) was used to record the running times, distances and traces in the exercise group, and WeChat software (Shenzhen, China) was used to monitor the daily step number in the control group.

### 4.3 Outcomes and Follow‐Up

#### 4.3.1 Primary Outcome

The primary outcome was the self‐assessed severity of perceived anxiety symptoms, obtained using the SAS, a clinically well‐established psychiatric assessment scale [27]. This outcome was assessed at baseline, at the completion of the exercise intervention (week 8), and at the 3‐month postintervention follow‐up (week 20).

#### 4.3.2 Secondary Outcomes

Participants' depression levels, which served as the secondary outcome, were measured by the self‑rating depression scale (SDS) [68]. This outcome was assessed at baseline, immediately after the 8‑week exercise program, and again 3 months later.

### 4.4 Isolation of Serum Proteomes and Proteomics

We profiled and compared the serum proteomes of 32 participants from the exercise group and 33 from the control group. First, we collected blood samples at the baseline and one day post‑intervention (both between 7:00 and 9:00 a.m.). The samples were subsequently spun at 3000 × *g* for 30 min at 4°C to obtain plasma. We depleted high‑abundance proteins using a Pierce Top 12 Abundant Protein Depletion Spin Columns Kit (Thermo Scientific, USA). A BCA kit (Beyotime, China) was used to quantify the protein concentration, according to the manufacturer's guidelines. After reduction with 5 mm dl‑dithiothreitol (56°C, 30 min) (Sigma‒Aldrich, USA) and alkylation with 11 mm iodoacetamide (room temperature, dark, 15 min) (Sigma‒Aldrich, USA), the protein samples were diluted with 100 mm tetraethylammonium bromide (Sigma‒Aldrich, USA) to reduce the urea concentration to <2 m. Trypsin was then added at a 1:50 ratio for the first overnight digestion, and a second aliquot of trypsin (1:100 ratio) was applied for a subsequent 4‑h incubation. We resolved the peptides on an EASY‑nLC 1200 UHPLC system after dissolving them in mobile phase A. The separation employed a two‑solvent system: A (0.1% FA in 2% ACN/water) and B (0.1% Formic Acid (FA) in 90% Acetonitrile (ACN)/water). The separated peptides were then ionized via a nanoelectrospray ionization (NSI) source and analyzed on an Orbitrap Exploris 480 mass spectrometer (Thermo Fisher Scientific). LC‒MS/MS analysis was performed by Jingjie PTM BioLab (Hangzhou, China). The resulting MS/MS spectra were processed using Proteome Discoverer software (v2.4.1.15). The tandem mass spectra were searched against the Homo_sapiens_9606_PR_20210721.fasta database (78120 entries) appended with a reverse decoy sequence. The mass spectrometry proteomics data have been deposited to the ProteomeXchange Consortium via the PRoteomics IDEntifications Database (PRIDE) [69] partner repository (http://www.ebi.ac.uk/pride) with the dataset identifier PXD067565.

### 4.5 Proteomic Analyses

The raw LC‑MS data were initially matched against the database, and the resulting identifications were converted into intensity matrices following normalization. In this step, the raw intensity was corrected for sample/batch effects to obtain the normalized protein intensity. Subsequently, the normalized intensity (*I*) underwent centralization to generate the relative quantitative value (*R*). Differential analysis was conducted using pairwise group comparisons, where the fold change (FC) for each protein was defined as the quotient of the mean intensities between the two sample groups. Student's *t*‑test was used to compare each protein's relative quantitative values between the two groups, with significance set at *p* < 0.05. Log2 transformation was applied to the relative quantitative values to make the data more compatible with the normality assumption of the *t*‑test. The criteria for differential expression were set at *p* < 0.05 and a fold change of >1.5 (upregulation) or <1.5 (downregulation). GO, KEGG pathway, and protein domain enrichment analyses were performed on the DEPs identified in each comparison group. For each functional term, enrichment significance was evaluated using Fisher's exact test, adopting a threshold of *p* < 0.05. The raw sequence data were uploaded to the PRIDE and are available via the following link: https://www.ebi.ac.uk/pride/login.

### 4.6 Machine Learning Workflow

All analyses for (i) selecting candidate biomarkers and (ii) constructing predictive classification models on the basis of MS proteome expression profiles were implemented in Python with the scikit‐learn package. The complete workflow consisted of the following steps.

1. *Data preprocessing*: Proteomic expression matrices were preprocessed by (i) removing proteins with a high proportion of missing values, (ii) imputing any missing values using the *k*‐nearest neighbors (KNN) algorithm to generate a complete feature matrix, and (iii) applying normalization to ensure comparability across samples. After preprocessing, each sample was represented by a feature vector comprising 1443 protein expression variables.
2. *Dataset partitioning*: To enhance generalizability, the dataset was split via stratified sampling into a training set and an independent test set while ensuring comparable class proportions across subsets.
3. *Class imbalance handling (training only)*: Potential class imbalance in the training set was addressed via oversampling (e.g., SMOTE and SVM‐SMOTE [when applicable]).
4. *Feature ranking*: In the training set, univariable feature ranking was performed using variance‐based statistical testing (e.g., analysis of variance (ANOVA)/variance test). For each protein feature, the association with the class label (exercise vs. control) was quantified, yielding a *p*‐value. Features were then ranked by ascending *p*‐values, where smaller *p*‐values indicate stronger discriminative evidence.
5. *IFS and model construction*: Incremental feature selection was implemented by constructing feature subsets containing the top *i*‐ranked proteins, with *i* being increasing iteratively. For each candidate subset size, a voting ensemble was trained and evaluated using tenfold cross‐validation within the training cohort. The ensemble comprises three base learners: a LR algorithm, an SVM, and an RM algorithm. Each base learner generates class probabilities, which are combined using a weighted voting/probability aggregation strategy; the final class label is assigned on the basis of the maximum aggregated score.

Model selection during IFS was guided by Matthews’ correlation coefficient (MCC). The optimal feature subset was defined as the subset that yielded the maximum MCC; a locally optimal subset was also identified within the top region of the IFS performance profile.

1. *Model evaluation*: Receiver operating characteristic (ROC) analyses were performed using the selected optimal (and locally optimal) feature subsets to assess the predictive performance of the model in the training set and independent test set. The selected biomarkers were further evaluated in downstream analyses to support their robustness and biological relevance.
2. *Prevention of data leakage and overestimation*: To mitigate potential data leakage and performance overestimation, feature ranking, incremental feature selection, class‐balance processing (SMOTE/SVM‐SMOTE), model training, and model selection were conducted exclusively in the training cohort and/or each cross‐validation fold. The independent test cohort was held out and used only once for the final evaluation.

### 4.7 Collection of Blood Samples From Patients With Anxiety Disorders

This study was approved by the Ethics Committee of the First Affiliated Hospital of Xi'an Jiaotong University, China (Approval ID: XJTU1AF2022LSK‐249). The study obtained written informed consent from all enrolled individuals. Blood samples were taken from anxious patients and unmedicated healthy controls within a 1‑week period. All the collections were performed between 7:00 and 9:00 a.m., and all the participants completed the GAD‐7 assessment. The clinical diagnosis of anxiety disorder was established following completion of a structured clinical interview and the Hamilton anxiety rating scale (HAM‐A).

### 4.8 Animals

A total of 210 C57BL/6 mice aged 6 weeks were procured from Beijing Vital River Laboratory Animal Technology Co., Ltd. The mice were housed in a controlled environment with a 12‐h light/dark cycle (dark hours: 20:00–8:00), a temperature of 21°C –23°C and a humidity level of 50% ± 10%. Food and water were provided ad libitum. All the animal care procedures adhered to the Animal Welfare Act, and the experimental protocols were approved by the Animal Care and Use Committee of Xi'an Jiaotong University (Approval ID: XJTUAE2023‐2337).

### 4.9 Treadmill Exercise

Treadmill exercises were performed over seven consecutive days, during which the mice ran daily for 15, 30, 60, 90, or 120 min at a constant speed of 10 m min^−1^; each 15 min of running was followed by a 1‐min rest period. Control mice were placed on a stationary treadmill daily for 30 min and allowed free movement. Tissues were collected 30 min after the final running session.

### 4.10 Voluntary Exercise

All exercise training were carried out in running‑wheel activity cages. The activity cages housed exercising mice in randomly assigned pairs with freely rotating wheels, whereas the sedentary counterparts were paired in cages with immobilized wheels serving as enrichment. Running distance, elapsed time, maximum speed, and average speed were recorded daily using a monitoring system composed of a magnetic reed switch (attached to a digital odometer from Sunding, China) and a magnet fixed to the 11‑cm‑diameter running wheel (Carno, China).

### 4.11 The CIS Model

The mice were subjected to 2 h of CIS each day for 10 consecutive days via disposable restrainers.

### 4.12 In Vivo EdU Pulses

Beginning on day 26 of the intervention, the mice were injected intraperitoneal (i.p.) with EdU (50 mg kg^−1^, MCE, China) once a day for three consecutive days to label proliferating cells.

### 4.13 Cy5.5 Labeling of rRPLP2

The near‑infrared fluorescent dye Cy5.5 was covalently attached to the recombinant RPLP2 protein through a standard chemical coupling procedure. Briefly, purified rRPLP2 was incubated with a tenfold molar excess of Cy5.5–*N*‐hydroxysuccinimide (NHS) ester (Biolite, China) in 0.1 m NaHCO_3_ (pH 8.5) for 2 h at 25°C in the dark. The reaction mixture was then passed through a pre‐equilibrated desalting column to remove unreacted free dye, yielding purified labeled protein (rRPLP2–Cy5.5). The mice received tail vein injections of the rRPLP2–Cy5.5 conjugate at a dose of 1.5 mg kg^−1^. At 20 h postinjection, the mice underwent transcardial perfusion using ice‑cold phosphate‐buffered saline (PBS) to thoroughly flush unbound fluorescent agent from the vasculature. The brains were rapidly harvested, embedded in optimal cutting temperature (OCT) compound, snap frozen, and 16‑µm‑thick coronal sections were obtained via freezing microtome sectioning.

### 4.14 rRPLP2 Treatment

To mimic the exercise‐induced increase in circulating RPLP2 levels, the mice received tail vein injections of rRPLP2 at a dose of 1.5 mg kg^−1^ (Ruihua, China). The injections were initiated on day 1 of the experimental intervention and were administered every other day for a total of 14 injections.

### 4.15 Behavioral Assays

#### 4.15.1 Elevated Plus‐Maze Test

The elevated plus‐maze test is widely used to assess anxiety‐like behaviors in animals. The maze consisted of two open arms (30 cm × 8 cm), two closed arms with walls (30 cm × 8 cm × 25 cm), and a central platform (8 cm × 8 cm) elevated 1 m above the ground. The mice were first set on the central platform, oriented toward one open arm, and subsequently given 5 min of unrestricted exploration in the maze. Entries into the open arms were tracked and recorded via SMART v.3.0 software. Prior to testing, all animals were habituated to the testing environment for 2 h. The maze was cleaned with 75% ethyl alcohol after each trial to eliminate odor interference.

#### 4.15.2 Light/Dark Box

The light/dark box was defined as an open field apparatus (45 cm × 45 cm × 45 cm) consisting of two equal compartments separated by a gate. One compartment is brightly lit, while the other is dark. The mice were individually placed in the center of the brightly lit compartment, facing away from the gate, and allowed 5 min of free exploration between the two chambers. SMART v.3.0 software was used to track and record the number of compartment transitions and the duration spent in the dark chamber. Prior to testing, all the mice were acclimatized for 2 h in the testing room. The open field was cleaned with 75% ethyl alcohol after each trial to eliminate odor interference.

### 4.16 Microdissection of Dentate Gyrus Tissue

For DG‐specific tissue collection, the brains were excised and immediately placed in ice‐cold buffer. The hippocampus was dissected and placed under a stereomicroscope on an ice‐cold surface, where the DG was identified according to established anatomical landmarks. The hippocampal fissure was used as the visual boundary to separate the DG from Ammon's horn. The DG blade and region containing the hilus were carefully separated from the surrounding Cornu Ammonis (CA) regions using a fine microdissection scalpel. Dissected DG tissues from the hippocampus were collected in RNase‐free tubes, rapidly frozen and kept at −80°C for subsequent RNA or protein isolation.

### 4.17 Protein Extraction and Immunoblotting

Fresh cells and tissues were sonicated in a buffer solution containing phosphatase and protease inhibitors (MCE, HY‐K0021 and HY‐K0011, respectively) for preparing a cell suspension. The samples were then centrifuged at 12,000 × *g* for 20 min at 4°C. Serum samples were processed with an Albumin Depletion Kit for Serumor Plasma (Beyotime, P2293M) following the manufacturer's protocol. Protein concentrations were quantified with a BCA kit (Beyotime, P0011) according to the manufacturer's protocol. After denaturation in loading buffer (100°C, 10 min), the proteins were electrophoresed on Sodium Dodecyl Sulfate‐Polyacrylamide Gel Electrophoresis (SDS‐PAGE) and electroblotted onto Polyvinylidene Difluoride (PVDF). The membranes were then blocked with 5% skim milk for 2 h and probed with primary antibodies at 4°C overnight. The next day, after washing with 0.01% Tris‐Buffered Saline with Tween‐20 (TBST), the membranes were incubated with secondary antibodies for 2 h. The immunoreactive signals were captured with a Clinx imaging system (Clinx, China), and band intensities were measured by ImageJ (NIH, Bethesda, USA). Details of all antibodies used are available in Table S4.

### 4.18 Tissue Processing and Immunofluorescence Staining

Following deep anesthesia and transcardial saline perfusion, the right cerebral hemisphere, muscle, and other organs and tissues of interest were snap‐frozen in liquid nitrogen and kept at −80°C for immunoblotting and qPCR, while the left hemisphere underwent postfixation in 4% paraformaldehyde (48 h, 4°C) and cryoprotection in 30% sucrose (48 h). Hippocampal coronal sections (16 or 30 µm) were cut on a freezing microtome and stored in cryoprotective medium (40% PBS, 30% glycerol, and 30% ethylene glycol) at −20°C. The muscle samples were blotted with absorbent paper to remove excess blood and fluid, embedded in OCT compound (Sakura, 4583), frozen in liquid‐nitrogen–cooled isopentane, and stored at −80 °C; transverse sections (10 µm thick) were also obtained. Prior to staining, the muscle sections were fixed with 4% paraformaldehyde for 20 min. The sections were subsequently washed in PBS 3–5 times for 10 min each. The sections were blocked with PBS containing 5% goat serum and 0.3% Triton X‐100 for 1 h at room temperature, followed by incubation with primary antibody overnight at 4°C, and then with fluorophore‑conjugated secondary antibodies (2 h, room temperature), with DAPI counterstaining (Sigma‒Aldrich, MO, USA). Antibody details are provided in Table S4. Confocal imaging was performed on FV4000 (Olympus, Tokyo, Japan) and AX/AX R (Nikon, Tokyo, Japan) microscopes.

### 4.19 Image Acquisition, Processing, and Analysis

To quantify neurogenesis‐specific markers in the DG, every sixth section (10–12 sections per animal) spanning the entire hippocampus (from bregma −1.5 mm to bregma −3.5 mm) was selected for analysis; values were obtained at the mouse level by averaging the values from all the sections from each mouse. Double‑blinded investigators performed all counting procedures using ImageJ/FIJI software. Specifically, fluorescent cells (BrdU+, DCX+, and BrdU+DCX+) were counted by examining all optical sections in confocal *z*‑stacks without maximal projection, following a previously reported method [70]. Only cells displaying overlapping signals in three or more consecutive *z*‑planes were considered double positive. The DG granule cell layer was delineated according to DAPI staining and stereotaxic coordinates from the mouse brain atlas. All the sampling grids were fully surveyed without skipping during the careful counting process. For each marker of interest, positive cells were quantified within the defined DG region. Specific sample sizes (*n*, numbers) for each quantification are provided in the figure legends.

Dendritic arborization was assessed in retrovirus‑labeled DG neurons imaged on a confocal microscope (FV4000; Olympus, Tokyo, Japan) from 100‑µm floating sections, with Sholl parameters and total dendritic length quantified in ImageJ. A minimum of 10–15 cells per group (from ≥3 animals) were included, with group sizes specified in figure legends. Spine analysis was performed on dendritic segments (50–150 µm from soma) of Lentivirus (LV)‑EGFP‑expressing DG neurons using Imaris software (Bitplane, Zürich, Switzerland). Spine density was expressed as spines µm^−1^, and spines were assigned to morphological classes according to length and head‑to‑neck diameter ratio: stubby (<1 µm and ratio <1.5), thin (≥1 µm and ratio <1.5), and mushroom (ratio ≥1.5, length unrestricted) [71].

### 4.20 ELISA

Blood samples were placed in procoagulant tubes. After incubation at room temperature for 2 h, the samples were centrifuged at 3500 × *g* for 20 min. The serum from the upper layer was collected for further analysis, and a human RPLP2 ELISA kit (Jianglai Biotechnology, JL16572) was used to determine the RPLP2 levels of the groups. Mouse sera were obtained in serum separator tubes, left to clot for 2 h at room temperature, and subsequently spun for 20 min at 3500 × *g*. The serum RPLP2 levels of the mice were measured with a mouse 60S RPLP2 ELISA kit (Signalway Antibody LLC, EK20530) according to the manufacturer's protocol.

### 4.21 RT‒qPCR

The expression levels of RPLP2 in various mouse tissues were assessed via RT‒qPCR. Total RNA was purified from the mouse tissues via TRIzol reagent, and complementary DNA (cDNA) was prepared using a reverse transcription kit (Monad, MR05001) as per the manufacturer's guidelines. RT‒qPCR was then run on a Bio‐Rad CFX Real‐Time PCR System (Bio‐Rad, USA) and MonAmp ChemoHS qPCR Mix (Monad, MQ00401) using CTACGTCGCCTCTTACCTGC as the forward primer and ACTCCTCGGACTCCTCCTTC as the reverse primer. Gene expression levels were calculated via the 2^−∆∆^ *^Ct^* method and normalized to those of β‐actin.

### 4.22 Ex Vivo Electrophysiology

The brains of isoflurane‐anesthetized mice were rapidly extracted and subjected to coronal sectioning (300 µm) on a vibrating microtome (VT1200 S, Leica) in cold (0°C–4°C) choline dissection solution containing 110 mm choline chloride, 2.5 mm KCl, 1.25 mm NaH_2_PO_4_, 0.5 mm CaCl_2_, 10 mm MgSO_4_, 25 mm NaHCO_3_, 11 mm d‐(+)‐glucose, 20 mm 4‐(2‐hydroxyethyl)‐1‐piperazineethanesulfonic acid (HEPES), 5 mm sodium ascorbate, and 3 mm sodium pyruvate saturated with 95% O_2_ and 5% CO_2_ (pH 7.4, 308–312 mOsm). After 10 min of incubation at 30°C, the slices were transferred to 95% oxygen/5% carbon dioxide‐saturated artificial cerebrospinal fluid (aCSF) containing 126 mm NaCl, 26 mm NaHCO_3_, 10 mm NaCl, 26 mm NaHCO_3_, 10 mm d‐(+)‐glucose, 2.5 mm KCl, 1.25 mm NaH_2_PO_4_, 1.2 mm MgCl_2_, and 2 mm CaCl_2_ (pH 7.4, 305–315 mOsm) and maintained at room temperature. The slices containing the DG were placed in the recording chamber and continuously perfused with aCSF (3–4 mL min^−1^) at room temperature. Patch pipettes were maintained at a resistance of 4–7 MΩ and filled with an intracellular solution containing 130 mM K‐gluconate, 20 mm KCl, 10 mm HEPES, 4 mm Mg‐ATP, 0.3 mm Na‐Guanosine Triphosphate (GTP), 10 mm disodium phosphocreatine and 0.2 mm Ethylene Glycol‐bis(2‐aminoethyl ether)‐N,N,N',N'‐Tetraacetic Acid (EGTA) (pH 7.4, 290–295 mOsm). For current–clamp recording, action potentials from newly formed GFP+ neurons in the DG were recorded following the injection of 0–200 pA current at a holding potential of −70 mV. All the recordings were conducted via an upright Olympus microscope (BX51WIF, Olympus, Tokyo, Japan). Signals were obtained through an integrated patch‐clamp amplifier (Sutter Instrument, USA) and acquired with IGOR Pro 8 software (WaveMetrics, USA). Series resistance was monitored during recording, and data were discarded for those altered by >20%.

### 4.23 Viral Injections

Mice were anesthetized with isoflurane and fixed to a brain stereotaxic apparatus (RWD Life Science, China). For the hippocampal RPLP2 knockdown, pAAV2/8‐U6‐shRNA(RPLP2)‐CMV‐EGFP‐WPRE (2.5 × 10^10^ vg mL^−1^) or pAAV2/8‐U6‐shRNA(NC)‐CMV‐EGFP‐WPRE (2.5 × 10^10^ vg mL^−1^) virus was bilaterally injected into the DG at a volume of 500 nL and a speed of 100 nL min^−1^ at the following coordinates relative to bregma: Anterior‐Posterior (AP) = −2.00 mm, Medial‐Lateral (ML) = +1.50 mm, and Dorsal‐Ventral (DV) = −2.15 mm. To label newly formed neurons in the DG, pRetro‐EF1‐EGFP‐3xFLAG‐WPRE (5.02 × 10^8^ g mL^−1^) virus was bilaterally injected at a volume of 1 µL and a speed of 0.05 µL min^−1^. Following the conclusion of the injection procedure, the nanoliter syringe was retained in situ for an additional 10 min prior to removal. The skin was subsequently stitched and cleansed with iodophors. For the muscle virus injections, the hair over the hindlimb muscles was shaved and disinfected with iodine and alcohol. The needle was then inserted at a 45° angle into the muscle at a depth of approximately 3–5 mm. The following viral vectors were slowly injected into the hindlimb muscles of the mice: pAAV2/8‐U6‐shRNA(RPLP2)‐CMV‐EGFP‐WPRE (2.5 × 10^10^ vg mL^−1^), pAAV2/8‐U6‐shRNA(NC)‐CMV‐EGFP‐WPRE (2.5 × 10^10^ vg mL^−1^), SCAAV2/MyoAAV 2A‐mhck7‐IGK‐RPLP2‐HA‐WPREs (5 × 10^11^ vg mL^−1^), and SCAAV2/MyoAAV 2A‐mhck7‐HA‐WPREs (5 × 10^11^ vg mL^−1^). The control virus lacked a target gene conjugated to the HA tag—a small peptide tag used primarily for protein detection that does not affect the ability of the fusion target proteins to cross the BBB—and served as an empty vector control. Each hindlimb muscle was injected at eight sites (2 µL per site). After the needle was withdrawn, gentle pressure was applied to achieve hemostasis. Injections were performed every other day for a total of four administrations. The relevant procedures were performed following a 2–4‐day recovery period.

### 4.24 Neural Stem Cell Primary Cultures

NSPCs were isolated from mouse brain tissues at embryonic days 14–17 with minor modifications to a published protocol [72, 73]. After meningeal removal, the tissue was minced (1‑mm^3^ pieces) and trypsinized (0.25%, 20 min, 37°C; Gibco, 25200072), followed by centrifugation at 200 × *g* for 5 min. The cell suspension was cultured in serum‐free Dulbecco's modified Eagle's medium (DMEM)/F12 medium (Gibco, c11330500BT) supplemented with 20 µg L^−1^ basic fibroblast growth factor (MCE, HY‐P70439), 20 µg L^−1^ epidermal growth factor (MCE, HY‐P7067), 2% B27 (Gibco, 17504044), 30% glucose and 1.83 mg mL^−1^ heparin. The samples were subsequently passaged with accutase (Millipore, 00‐4555‐56) every 2–3 days. After passage 3, the neuroglobin enzymes were separated into single cells for downstream applications. For the in vitro differentiation experiments, the NSPCs were centrifuged at 200 × *g* for 5 min to collect P3‐generation NSPCs, which were subsequently resuspended in differentiation medium containing 25% DMEM/F12 (Gibco, c11330500BT), 75% neurobasal medium (Gibco, 21103049), 1% B27 (Gibco, 17504044), and 0.5% N2 (Gibco, 17502048). The cell suspension was seeded at a density of 5 × 104 cm^−2^ into a 24‐well plate prepacked with Poly‐L‐Lysine (PLL) (Sigma, p1399). For the muscle cell–conditioned coculture experiments, 5% concentrated muscle supernatant or muscle supernatant without the RPLP2 protein from the Pierce Classic Magnetic IPCo‐IP kit (Thermo Fisher Scientific, 88804) was added to the differentiation medium. For the RPLP2 intervention experiment, 100 ng mL^−1^ RPLP2 was added to the differentiation medium. The cells were subjected to immunofluorescence analysis after 7 days. During differentiation, the culture medium was supplemented with the following for subsequent RNA‐seq analyses: pAAV‐U6‐shRNA(RPLP2)‐CMV‐EGFP‐WPRE (2.5 × 10^1^ ^2^ vg mL^−1^), pAAV‐U6‐shRNA(NC)‐CMV‐EGFP‐WPRE (2.5 × 10^1^ ^2^ vg mL^−1^), rRPLP2 (100 ng mL^−1^), or PBS.

### 4.25 C2C12 Cell Cultures

Mouse myoblast C2C12 cells (ATCC CRL1772) were cultured in 75 cm^2^ flasks in DMEM (Gibco, C11995500BT) supplemented with 10% heat‐inactivated fetal bovine serum (Cellmax, SA211.01) and 1% penicillin/streptomycin (Cellmax, CPS101.05). The cells were incubated at 37°C under a humidified 5% CO_2_ atmosphere, with the culture medium refreshed every 2–3 days. At 80%–90% confluence, the cells were passaged using 0.25% trypsin. To induce differentiation, the growth medium was switched back to DMEM (Gibco, C11995500BT) supplemented with 2% horse serum (Gibco, 16050130), which was replaced every 1–2 days. For HA tagging, cells were placed in 6‐well plates and transduced with scAAV‐mhck7‐IGK‐RPLP2‐HA‐WPRE virus (40 µL, 5×10^1^ ^1^ vg mL^−1^) and 8 µg mL^−1^ polybrene. Replacement of the viral medium with fresh growth medium was performed at 24–48 h.

### 4.26 Ribosome Extraction

The cultured, 1 × 10^7^ differentiated NSPCs were centrifuged at 1000 × *g* for 5 min at 4°C for cell collection. Ribosomes were extracted from differentiated NSPCs using a ribosome isolation kit (Solarbio Science & Technology, EX1380).

### 4.27 Puromycin Incorporation Assays

To assess nascent protein synthesis, cells were exposed to rRPLP2 and then incubated with puromycin (Sigma, P9620) (10 µg mL^−1^) for 5 min. Cell were washed with ice‐cold PBS, lysed, and analyzed via immunoblotting using an antipuromycin antibody.

### 4.28 RNA Extraction, Library Construction, and Sequencing

Total RNA was isolated with TRIzol Reagent (Thermo Fisher, 15596018) per the supplier's protocol, and its integrity (RIN ≥ 7.0) and concentration were determined using an Agilent Bioanalyzer 2100 with an RNA 6000 Nano Kit (Agilent, 5067‐1511). From 5‑µg aliquots of total RNA, mRNA was enriched by two rounds of Dynabeads Oligo (dT) selection (Thermo Fisher) and fragmented at 94°C for 6 min (±1 min) with the Mg^2^^+^‐based fragmentation (NEBNext Magnesium RNA Fragmentation Module, NEB E6150). The fragmented RNA was converted to cDNA using SuperScript II Reverse Transcriptase (Invitrogen, 1896649) for first‑strand synthesis, followed by second‑strand synthesis with deoxyuridine triphosphate (dUTP) incorporation using *Escherichia coli* DNA Polymerase I (NEB M0209), RNase H (NEB M0297), and dUTP (Thermo Fisher, R0133). The cDNA ends were A‐tailed and then ligated to dual‐indexed adapters with T‐overhangs. Size selection (AMPure XP beads) yielded fragments averaging 300 ± 50 bp in length. Uracil‐containing strands were digested with Uracil‐DNA Glycosylase (UDG) (NEB M0280). Libraries were PCR‐amplified (8 cycles: 98°C/15 s, 60°C/15 s, 72°C/30 s), and PE150 was sequenced on an Illumina NovaSeq 6000 (LC‐Bio, Hangzhou) following standard protocols.

### 4.29 RNA‐Seq Analysis

A cDNA library was constructed from pooled NSPC RNA and sequenced on an Illumina NovaSeq 6000 platform using the paired‑end (2 × 150 bp) mode, yielding approximately 1 million reads per sample. Raw reads containing adapters or low‑quality bases were filtered with Cutadapt (v1.9) to obtain clean reads, and sequence quality (Q20, Q30, and GC content) was verified with FastQC (v0.11.9). A total of 28.7 Gb of cleaned paired‑end reads was generated. The raw data have been deposited in the NCBI Gene Expression Omnibus (GEO) under accession number: https://www.ncbi.nlm.nih.gov/geo/query/acc.cgi?acc = GSE305841. Clean reads were aligned to the human reference genome using HISAT2 (v2.2.1), and the mapped reads for each sample were assembled with StringTie (v2.1.6) under default parameters. All sample‑specific transcriptomes were merged into a comprehensive reference using gffcompare (v0.9.8). Transcript abundance was quantified with StringTie and Ballgown, and expression levels were normalized as fragments per kilobase of transcript per million mapped reads (FPKM). Differential expression analysis was performed with DESeq2 (for group comparisons) and edgeR (for pairwise sample comparisons), with statistical significance defined as false discovery rate (FDR) < 0.05 and |fold change| ≥ 2. Differentially expressed genes were subsequently subjected to GO and KEGG enrichment analyses. All bioinformatic analyses were conducted using the OmicStudio tools (https://www.omicstudio.cn/tool).

### 4.30 Statistical Analysis

#### 4.30.1 Statistical Methods for Population Experiments

Sample size determination was based on data from a previous investigation [74]. A total of 232 participants were required to detect a predicted three‐point difference in the SAS score when considering a standard difference in scores of 10 and a power of at least 80% to achieve a two‐sided significance level of 5%. In this study, 272 participants were ultimately included to account for potential missing samples. Between group comparisons of baseline variables were performed with *t*‑tests (continuous) and *χ* ^2^ tests (categorical). Differences in primary and secondary outcomes were evaluated with a mixed‑effects model for repeated measures (MMRM), with group (running vs. nonrunning) and time (baseline vs. week 8/week 20) as individual fixed effects. The relationship between the interaction and outcome was assessed to test whether the outcomes varied by exercise intervention. Correlations between repeated measurements from the same participant were modeled with an autoregressive covariance matrix. Missing values in the human study were imputed using the KNN method. At enrollment, 136 participants were included in each group; data imputation was performed to retain 136 participants per group in the longitudinal analyses. Missing values were limited to SAS and SDS scores at the follow‐up assessments. After the 4‐week intervention, 127 participants in the exercise group and 131 participants in the control group had completed the assessment; therefore, the SAS and SDS scores were imputed for nine participants in the exercise group and five participants in the control group. At the 20‐week follow‐up, 123 participants in the exercise group and 128 participants in the control group had completed the assessment; therefore, the SAS and SDS scores were imputed for 13 participants in the exercise group and 8 participants in the control group. In total, 70 values were imputed, including 35 SAS values and 35 SDS values. No missing values were present for group assignment or baseline enrollment information. Correlations between SAS scores and serum protein abundance values were assessed using Spearman correlation analysis, a rank‐based nonparametric test that does not require normally distributed data, as most serum protein abundance values did not satisfy the assumption of normality according to normality testing. Analyses were performed in RStudio, version 1.4 (http://www.r‐project.org), and IBM SPSS Statistics, version 23. All analyses were performed on an intention‐to‐treat (ITT) basis, and a two‐sided *p* < 0.05 was considered indicative of statistical significance.

#### 4.30.2 Statistical Methods for Mouse Experiments

Data are presented as mean ± standard error of the mean (SEM), with individual points in figures representing single mice, cells, segments, or independent experiments (*n* values in legends). GraphPad Prism 9 (GraphPad Software, CA, USA) was used for all statistical analyses. Normality and variance homogeneity were assessed by Shapiro–Wilk and F/Brown–Forsythe tests, respectively. For two‐group comparisons, unpaired *t*‐tests (with Welch's correction applied when variances were unequal) were used. For multigroup comparisons, normally distributed data with equal variances were analyzed by one‐way ANOVA or repeated‐measures ANOVA, followed by Tukey's multiple comparisons test. Normally distributed data with unequal variances were analyzed using Welch's ANOVA followed by Dunnett's T3 multiple comparisons test. Non‐normally distributed data were analyzed using the Kruskal–Wallis test followed by Dunn's multiple comparisons test. Sholl analysis was performed using repeated‐measures ANOVA. All values are presented as the mean ± SEM.

## Author Contributions

Concept and design: **Yan Li** and **Xiancang Ma**. Acquisition, analysis, and interpretation of the data: **Peiyu Luo**, **Wei Wu**, **Huan Peng**, **Dan He**, **Yuxi Guo**, **Xiaodan Wang**, **Li Ma**, **Yuhang Qin**, **Linlin Jing**, **Yifang Zhai**, **Lixia Zhuo**, **Ying Zhang**, **Yijie Guo**, **Erfei Zhang**, and **Wei Wang**. Drafting the manuscript: Yan Li, Xiancang Ma, Peiyu Luo, Wei Wu, and Huan Peng. Statistical analysis: **Boyue Zhao** and **Fangyao Chen**. Administrative, technical, or material support: Peiyu Luo, Wei Wu, Huan Peng, and Wei Wang.

## Conflicts of Interest

The authors declare no conflict of interest.

## Supporting information




**Supporting File 1**: advs76479‐sup‐0001‐SuppMat.docx.

**Supporting File 2**: advs76479‐sup‐0002‐TableS5.xlsx.

## Data Availability

The raw proteomics sequence data generated in this study have been submitted to the PRoteomics IDEntifications Database (PRIDE) and are accessible at the following website: https://www.ebi.ac.uk/pride/login. The raw transcriptomics sequence data generated in this study have been submitted to the NCBI Gene Expression Omnibus (GEO) datasets under the accession link: https://www.ncbi.nlm.nih.gov/geo/query/acc.cgi?acc = GSE305841. All other data and computational results supporting the findings of this study are available by request to the corresponding author.
