# Supplementary material for: RPLP2 Mediates the Beneficial Effects of Exercise on Stress Resistance Through Muscle–Brain Communication
Source: Adv Sci (Weinh). 2026 Jul 9;13(55):e76479. doi: 10.1002/advs.76479 (PMC13348651; doi:10.1002/advs.76479)
Supplement: Supplementary file 1 [file ADVS-13-e76479-s002.docx]

**SUPPLEMENTARY INFORMATION**

**RPLP2 mediates the beneficial effects of exercise on stress resistance through muscle–brain communication**

*Peiyu Luo, Wei Wu, Huan Peng, Dan He, Yuxi Guo, Boyue Zhao, Xiaodan Wang, Li Ma, Yuhang Qin, Yifang Zhai, Lixia Zhuo, Ying Zhang, Yijie Guo, Linlin Jing, Fangyao Chen, Erfei Zhang, Wei Wang, Xiancang Ma, and Yan Li*

**Contents:**

**Figures S1–S9**

**Tables S1–S4**

**Figure S1. Flowchart of participant assessment and group allocation in the randomized controlled trial.** ITT, intention-to-treat.

**Figure S2. Serum proteomics analysis and identification of exercise-induced biomarkers correlated with SAS scores and differential protein expression.** (A) Relative expression levels of differentially expressed proteins. The centerline within each box shows the median, and the top and bottom of each box represent the 75th and 25th percentile values, respectively. The upper and lower whiskers extend from the hinge to the largest and smallest values (n = 33 individuals in the nonrunning group; n = 31 individuals in the running group). Individual data points indicate outliers. (B–J) Spearman analysis of the SAS score with the protein levels of 67 DEPs revealed significant negative correlations with (B) HYOU1 (*r* = -0.333, *P* = 0.007), (C) RPLP2 (*r* = -0.367, *P* = 0.003), (D) PRSS2 (*r* = -0.276, *P* = 0.026), (E) ZG16 (*r* = -0.344, *P* = 0.005) and (F) PAPOLA (*r* = -0.312, *P* = 0.011) and significant positive correlations with (G) FLG (*r* = 0.322, *P* = 0.010), (H) RPL15 (*r* = 0.298, *P* = 0.017), (I) CHRDL2 (*r* = 0.373, *P* = 0.002), and (L) KRT4 (*r* = 0.311, *P* = 0.012) (n = 30‒33 individuals per group). (K) Top 30 protein features ranked by univariable significance (ascending *p* values) from variance-based class discrimination analysis. (L) Incremental feature selection (IFS) curves showing the Matthews correlation coefficient (MCC) and area under the ROC curve (AUC) as functions of the number of top *p* value–ranked features included in a voting classifier with tenfold cross-validation; the global peak MCC at 9 features defines the optimal subset. (M) Relative expression levels of proteins containing the optimal feature subset of 9 proteins as obtained through machine learning screening. (N) Exercise-induced changes in serum levels of RPLP2 (n = 85 individuals in the nonrunning group; n = 78 individuals in the running group). Statistical analysis was performed with simple linear regression or Student’s t test in GraphPad Prism 9. **P* < 0.05, ***P* < 0.01, ****P* < 0.001, *****P* < 0.0001.

**Figure S3.** **Mouse locomotion is not affected by chronic immobilization stress.** (A) ELISA-derived circulating RPLP2 protein levels after treadmill exercise (n = 7–8 mice per group). (B) Total distance run on the wheel (n = 20 mice). (C) Initial and final body weights of the mice (n = 20 mice per group). (D) Total distance traveled in the EPM test (n = 20 mice per group). (E) Representative immunoblotting bands of circulating RPLP2 protein levels (left) and relative protein levels (right) (n = 7 mice per group). Con: control, CIS: chronic immobilization stress, Exe: exercise, EPM: elevated plus-maze. Statistical analysis was performed with one-way ANOVA in GraphPad Prism 9. All values are presented as the mean ± SEM. ns: not significant, **P* < 0.05, ***P* < 0.01, *****P* < 0.0001.

**Figure S4.** **Mouse growth and locomotion are unaffected by hippocampal RPLP2 ablation.** (A) Representative brain slices showing RPLP2 expression in the DG of WT mice injected with either scramble or shRPLP2 virus. Scale bar = 250 μm. (B) Total distance run on the wheel after virus injection (n = 10 mice). (C) Initial and final body weights of the mice (n = 10‒18 mice per group). (D) qPCR-derived *RPLP2* gene expression in the hippocampal tissue of mice (n = 6 mice per group). (E) Total distance traveled in the EPM test (n = 10‒18 mice per group). (F) Representative immunoblotting bands of RPLP2 from hindlimb muscle tissue (left) and relative protein levels (right) (n = 10 mice per group). G) ELISA-derived circulating RPLP2 protein levels (n = 10 mice per group). (H) Representative immunoblotting bands of circulating RPLP2 protein levels (left) and relative protein levels (right) (n = 7 mice per group). Sed: sedentary, Exe: exercise, EPM: elevated plus maze, MUS: muscle. Statistical analysis was performed with one-way ANOVA in GraphPad Prism 9. All values are presented as the mean ± SEM. ns: not significant, **P* < 0.05, ***P* < 0.01.

**Figure S5. Muscle RPLP2 ablation does not affect body weight gain, locomotion, or neurogenesis in mice.** (A) pAAV-U6-shRNA(*RPLP2*)-CMV-EGFP was utilized for specific knockdown of *RPLP2* in the hindlimb muscles; a representative image shows EGFP expression. (B) Total distance run on the wheel after virus injection (n = 9–10 mice). (C) Initial and final body weights of the mice (n = 9‒10 mice per group). (D) qPCR-derived *RPLP2* gene expression in muscle tissue (n = 6 mice per group). (E) Representative immunoblotting bands of RPLP2 from muscle tissue (left) and relative protein levels (right) (n = 6 mice per group). (F) qPCR-derived *RPLP2* gene expression in multiple organs of the mice (n = 4 mice per group). (G) Total distance traveled in the EPM test (n = 9‒10 mice per group). (H) Representative immunofluorescence images of hippocampal slices stained for EdU and DCX. (I–K) Fold change (FC) of EdU^+^ (I), DCX^+^ (J), EdU^+^ and DCX^+^ cells (K) (n = 5 mice per group). White arrows indicate EdU/DCX double-positive cells. Scale bar = 20 μm. (L) qPCR-derived *RPLP2* gene expression in hippocampal tissue (n = 6 mice per group). (M) Representative immunoblotting bands of RPLP2 from hippocampal tissue (left) and relative protein levels (right) (n = 6 mice per group). Sed: sedentary, Exe: exercise, MUS: muscle, HIP: hippocampus, EPM: elevated plus maze. Statistical analysis was performed with one-way ANOVA in GraphPad Prism 9. All values are presented as the mean ± SEM. ns: not significant, **P* < 0.05.

**Figure S6.** **RPLP2 ablation in the hippocampus and muscle does not affect body weight gain or locomotion.** (A) Total distance run on the wheel after virus injection (n = 9‒14 mice per group). (B) Initial and final body weights of the mice (n = 9‒14 mice per group). (C) Total distance traveled in the EPM test (n = 9‒14 mice per group). (D) Representative immunoblotting bands of HA levels from multiple organs in mice. (E) Representative immunoblotting bands of serum HA levels in mice. (F) Representative immunofluorescence images of hippocampal slices stained for DCX and HA-Tag. Scale bar = 10 μm. Sed: sedentary, Exe: exercise, HIP: hippocampus, MUS: muscle, EPM: elevated plus maze. Statistical analysis was performed with one-way ANOVA in GraphPad Prism 9. All values are presented as the mean ± SEM. ns: not significant.

**Figure S7**. **RPLP2 in C2C12 cell medium promotes neurosphere proliferation and neuronal differentiation.** (A) Representative images of neurospheres under the indicated treatment conditions (left) and neurosphere diameters (right) (n = 25–39 neurospheres per group). Scale bars = 100 μm. (B) In target protein flow-through, the tagged protein binds to the nickel column, and the tag is cleaved to obtain the target protein. (C) Mice were injected intravenously with recombinant RPLP2 labeled with the cyanine 5.5 fluorophore. (D) Representative immunofluorescence images of hippocampal sections stained for NeuN and Cyanine 5.5. White arrows indicate NeuN^+^ and Cy5.5^+^ cells. Scale bar = 25 μm. (E–F) Representative immunofluorescence images of hippocampal sections stained for EEA1 (E) and Lamp1 (F) and colabeled with Nestin and Cyanine 5.5. Scale bar = 20 μm (left) and 5 μm (right). Sup: supernatant, w/o: without. Statistical analysis was performed with the two-sample t test in GraphPad Prism 9. All values are presented as the mean ± SEM; *****P* < 0.0001.

**Figure S8**. **RPLP2 in C2C12 cell medium promotes ribosome assembly in neurons.** (A) Representative images showing immunocytochemical localization of RPL24 (green) and RPS6 (red) after rRPLP2 stimulation (left). The colocalization tendency of RPL24 and RPS6 signals was estimated by Pearson’s R values (about threshold) shown on the right (n =7–8 independent experiments per group). White squares indicate the regions of interest (ROIs) shown in magnified growth cone images below. Scale bars = 3 μm. (B) Representative immunofluorescence staining of differentiated NSCPs cocultured in MCCM under different treatment conditions for RPS6 and DAPI expression (left) and plots of the pixel intensity along the white lines on the images (right); the colors are the same as those in the merged images. Scale bar = 10 μm. (C) Representative images of neurospheres after rRPLP2 administration (left) and neurosphere diameters (right) (n = 8 neurospheres per group). Scale bars = 100 μm. Statistical analysis was performed with one-way ANOVA in GraphPad Prism 9. All values are presented as the mean ± SEM; **P* < 0.05, ***P* < 0.01, ****P* < 0.001.

**Figure S9. Exogenous rRPLP2 can cross the blood‒brain barrier and promote the morphological maturation and plasticity of newborn hippocampal neurons in CIS-exposed mice.** (A) Representative immunoblotting bands of RPLP2 from hippocampal tissue (n = 6 mice per group). (B) Initial and final body weights of the mice (n = 9‒13 mice per group). (C) Long thin spine density of EGFP^+^ newborn neurons (n = 54–58 segments). (D) Stubby spine density of EGFP^+^ newborn neurons (n = 54–58 segments). (E) Summary of the resting potential of EGFP^+^ newborn neurons within the DG (n = 13 cells). (F–G) Fold change (FC) of EdU^+^ (F) and DCX^+^ (G) cells (n = 5 mice per group). (H) Total distance traveled in the EPM test (n = 9–13 mice per group). Con: control, CIS: chronic immobilization stress, Exe: exercise, EPM: elevated plus-maze. Statistical analysis was performed with one-way ANOVA in GraphPad Prism 9. All values are presented as the mean ± SEM; ns: not significant; ***P* < 0.01, ****P* < 0.001, *****P* < 0.0001.

| **Table S1. Baseline characteristics of the RCT participants** | | | |
| --- | --- | --- | --- |
| **Characteristics** | **Exercise group**  **(n = 136)** | **Control group**  **(n = 136)** | ***P* value** |
| **Sex**, no. (%) |  |  |  |
| Female | 90 (66.2) | 87 (64.0) | 0.703 |
| Male | 46 (33.8) | 49 (36.0) |  |
| **Education background**, no. (%) |  |  |  |
| Undergraduate | 85 (62.5) | 90 (66.2) | 0.527 |
| Graduate | 51 (37.5) | 46 (33.8) |  |
| **Age**, mean (SD), y | 22.8 (3.2) | 22.7 (3.2) | 0.831 |
| **Weight**, mean (SD), kg | 61.9 (11.8) | 60.7 (9.9) | 0.342 |
| **Height**, mean (SD), cm | 167.0 (7.8) | 166.2 (7.0) | 0.286 |
| **BMI**, mean (SD), kg/m^2^ | 22.1 (3.3) | 21.9 (2.8) | 0.503 |
| **SAS score**, mean (SD) | 30.2 (5.4) | 30.8 (6.0) | 0.422 |
| **SDS score**, mean (SD) | 33.0 (6.8) | 33.7 (6.9) | 0.379 |
| **Total weekly physical activity**,  mean (SD), MET-min/w | 1778.7 (1674.5) | 1535.8 (1306.2) | 0.218 |
| **Sitting per week**, mean (SD), min | 485.3 (598.9) | 398.5 (395.3) | 0.161 |

Abbreviations: BMI, body mass index; SAS, Self-rating Anxiety Scale; SDS, Self-rating Depression Scale.

| **Table S2. Primary and secondary outcomes with ITT analysis results** | | | | | | |
| --- | --- | --- | --- | --- | --- | --- |
|  | **Exercise group**  **(n = 136)** | **Control group**  **(n = 136)** | ***P* value** | | | |
|  |  |  | **Interaction term** | **Between-Groups** | **Within-Group^a^** | |
|  |  |  |  |  | Exercise | Control |
| **Primary outcome** |  |  |  |  |  |  |
| **SAS score**, mean (95% CI) |  |  |  |  |  |  |
| baseline | 30.2 (29.2, 31.2) | 30.8 (29.8, 31.7) | < 0.001 | 0.434 |  |  |
| week 8 | 28.5 (27.5, 29.5) | 31.3 (30.4, 32.3) |  | < 0.001 | 0.001 | 0.597 |
| week 20 (follow-up) | 29.1 (28.2, 30.1) | 30.1 (29.1, 31.1) |  | 0.177 | 0.191 | 0.759 |
| **Secondary outcomes** |  |  |  |  |  |  |
| **SDS score**, mean (95% CI) |  |  |  |  |  |  |
| baseline | 33.0 (31.8, 34.2) | 33.7 (32.5, 34.9) | 0.043 | 0.400 |  |  |
| week 8 | 31.9 (30.7, 33.1) | 34.5 (33.3, 35.7) |  | 0.003 | 0.133 | 0.389 |
| week 20 (follow-up) | 31.6 (30.4, 32.8) | 33.6 (32.4, 34.8) |  | 0.017 | 0.122 | 1.000 |

^a^Comparison with baseline at each time point. Abbreviations: ITT, intention-to-treat; SAS, Self-rating Anxiety Scale; SDS, Self-rating Depression Scale.

| **Table S3. Baseline characteristics of participants depicted in Fig. 1H and I** | | | |
| --- | --- | --- | --- |
| **Characteristics** | **Anxiety group**  **(n = 51)** | **Control group**  **(n = 54)** | ***P* value** |
| **Sex**, no. (%) |  |  |  |
| Female | 37 (72.55) | 39 (72.22) | 0.970 |
| Male | 14 (27.45) | 15 (27.78) |  |
| **Age**, mean (SD), y | 20.22 (3.56) | 18.54 (0.86) | 0.038 |
| **Weight**, mean (SD), kg | 60.59 (17.24) | 61.91 (11.01) | 0.223 |
| **BMI**, mean (SD), kg/m^2^ | 21.71 (5.42) | 21.11 (2.79) | 0.670 |
| **Body fat rate**, mean (SD) | 22.34 (7.47) | 21.19 (6.55) | 0.878 |
| **PHQ-9 score**, mean (SD) | 19.67 (4.70) | 3.07 (2.73) | <0.001 |
| **GAD-7 score**, mean (SD) | 12.90 (4.22) | 1.59 (1.54) | <0.001 |

Abbreviations: BMI, body mass index; PHQ-9: Patient Health Questionnaire-9; GAD-7: Generalized Anxiety Disorder-7.

| **Table S4. List of products** | | |
| --- | --- | --- |
| **Products** | **Species** | **Supplier** |
| Anti-RPLP2 | Rabbit | Abcam, Cat# ab154958 |
| Anti-DCX | Rabbit | Cell Signaling, Cat# 4604 |
| Anti-HA | Rabbit | Proteintech, Cat# 81290 |
| Anti-CD31 | Mouse | Proteintech, Cat# 66065 |
| Anti-NeuN | Mouse | Cell Signaling, Cat# 94403 |
| Anti-RPS6 | Mouse | Proteintech, Cat# 66886 |
| Anti-RPL24 | Rabbit | Proteintech, Cat# 17082 |
| Anti-Puro | Mouse | Sigma, Cat# MABE343 |
| Anti-EEA1 | Rabbit | Proteintech, Cat# 28347 |
| Anti-Lamp1 | Rabbit | Proteintech, Cat# 86337 |
| Anti-Nestin | Mouse | Invitrogen, Cat# MA1-81819 |
| Anti-GAPDH | Mouse | Yeasen, Cat# 30203ES50 |
| Alexa Fluor 488 anti-Rabbit  secondary antibody | Goat | Invitrogen, Cat#A11008 |
| Alexa Fluor 555 anti-Mouse  secondary antibody | Goat | Invitrogen, Cat#A21424 |
| Alexa Fluor 488 anti-Mouse  secondary antibody | Goat | Invitrogen, Cat#A11029 |
| Alexa Fluor 555 anti-Rabbit  secondary antibody | Goat | Invitrogen, Cat#31572 |
| Alexa Fluor 647 anti-Rabbit  secondary antibody | Goat | Invitrogen, Cat#31573 |
| HRP-conjugated Goat anti-Rabbit IgG(H+L) | Goat | Proteintech, Cat# SA00001-2 |
| HRP-conjugated Goat anti-Mouse IgG(H+L) | Goat | Proteintech, Cat# SA00001-1 |
| BeyoClick™ EdU Cell Proliferation Kit with AF555 |  | Beyotime, Cat# C0075S |
| Cyanine 5.5 monosuccinimidyl ester |  | Biolite, Cat# 151 |
| Phosphatase inhibitors |  | MCE, Cat# HY-K0021 |
| Protease inhibitors |  | MCE, Cat# HY-K0011 |
| Albumin Depletion Kit for Serumor Plasma |  | Beyotime, Cat# P2293M |
| BCA kit |  | Beyotime, Cat# P0011 |
| OCT |  | Sakura, Cat# 4583 |
| DAPI |  | Sigma-Aldrich, Cat# D9542 |
| Human Acidic Ribosomal Phosphoprotein P2 (RPLP2) ELISA Kit |  | Jianglai Biotechnology, Cat# JL16572 |
| Mouse 60S RPLP2 ELISA Kit |  | Signalway Antibody LLC, Cat# EK20530 |
| Reverse transcription kit |  | Monad, Cat# MR05001 |
| MonAmp™ ChemoHS qPCR Mix |  | Monad, Cat# MQ00401 |
| Trypsin |  | Gibco, Cat# 25200072 |
| Dulbecco's modified Eagle's medium (DMEM)/F12 medium |  | Gibco, Cat# c11330500BT |
| Basic fibroblast growth factor |  | MCE, Cat# HY-P70439 |
| Epidermal growth factor |  | MCE, Cat# HY-P7067 |
| B27 |  | Gibco, Cat# 17504044 |
| Accutase |  | Millipore, Cat# 00-4555-56 |
| Neurobasal medium |  | Gibco, Cat# 21103049 |
| N2 |  | Gibco, Cat# 17502048 |
| Poly-L-lysine |  | Sigma, Cat# p1399 |
| Pierce Classic Magnetic IPCo-IP Kit |  | Thermo Fisher Scientific, Cat# 88804 |
| Dulbecco’s Modified Eagle Medium |  | Gibco, Cat# C11995500BT |
| Heat-inactivated fetal bovine serum |  | Cellmax, Cat# SA211.01 |
| Penicillin/Streptomycin |  | Cellmax, Cat# CPS101.05 |
| Horse serum |  | Gibco, Cat# 16050130 |
| Ribosome isolation kit |  | Solarbio, Cat# EX1380 |
| Puromycin |  | Sigma, Cat# P9620 |
| TRIzol™ Reagent |  | Thermo Fisher, Cat# 15596018 |
| RNA 6000 Nano Kit |  | Agilent, Cat# 5067-1511 |
| SuperScript™ II Reverse Transcriptase |  | Invitrogen, Cat# 1896649 |
| E. coli DNA Polymerase I |  | NEB, Cat# M0209 |
| RNase H |  | NEB, Cat# M0297 |
| dUTP |  | Thermo Fisher, Cat# R0133 |
| UDG |  | NEB, Cat# M0280 |
